# Recent Contributions of Air- and Biomarkers to the Control of Secondhand Smoke (SHS): A Review

**DOI:** 10.3390/ijerph8030648

**Published:** 2011-03-01

**Authors:** Jacques J. Prignot

**Affiliations:** Département of Lung Diseases, Université Catholique de Louvain, 1, Place de l'Université, B 1348 Louvain-la-Neuve, Belgium; E-Mail: jacques.prignot@uclouvain.be; Tel.: + 32-81-221655

**Keywords:** secondhand smoke, air- and biomarkers, tobacco control

## Abstract

Since the publication of the US Surgeon General Reports in 1996 and 2006 and the report of the California Environmental Protection Agency in 1999, many reports have appeared on the contribution of air and biomarkers to different facets of the secondhand smoke (SHS) issue, which are the targets of this review. These recent studies have allowed earlier epidemiological surveys to be biologically validated, and their plausibility demonstrated, quantified the levels of exposure to SHS before the bans in various environments, showed the deficiencies of mechanical control methods and of partial bans and the frequently correct implementation of the efficient total bans. More stringent regulation remains necessary in the public domain (workplaces, hospitality venues, transport sector, *etc.*) in many countries. Personal voluntary protection efforts against SHS are also needed in the private domain (homes, private cars). The effects of SHS on the cardiovascular, respiratory and neuropsychic systems, on pregnancy and fertility, on cancers and on SHS genotoxicity are confirmed through experimental human studies and through the relationship between markers and prevalence of disease or of markers of disease risk.

## 1. Introduction

The main issues of secondhand smoke (SHS) have been extensively addressed in two successive reports of the US Surgeon General [1,2] and in the report of the California Environmental Protection Agency [3].

Early publications about the health consequences of SHS (also referred to as Environmental Tobacco Smoke [ETS]) were mainly epidemiological surveys through questionnaires for general or specific populations. Their value is limited, as the respondents’ answers may (voluntarily or not) be incorrect and the large numbers of factors influencing the prevalence, intensity and duration of SHS exposure cannot be correctly estimated in a questionnaire. On the other hand, measurement of air- and biomarkers of SHS exposure is more quantitative and can complement and validate the results of the questionnaire surveys.

Recent contributions of air- and biomarkers to different facets of the SHS issue are reviewed in this paper. Medline was searched from 2005 with the keywords “SHS” and “air” or “atmospheric markers” and “SHS and biomarkers”. The full collection of Tobacco Control and Nicotine & Tobacco Research was examined from 2005 onward, and some references from bibliographies were also recorded. The Cochrane Review 2010 [4] was another source of information. To produce an overview compatible with the length of a review article, some publications were summarized and others had to be set aside.

Experimental studies on cells, germs and animals, transplacental foetus exposure during pregnancy and the economic consequences of smoking bans were excluded, as were the health effects of SHS estimated from risk models.

## 2. Methodological Issues in Markers

### 2.1. Atmospheric Markers

Among the potential atmospheric markers of SHS, carbon monoxide (CO) is usually excluded due to its poor specificity, although its non-tobacco-specific origin in closed environments is easily detected. In ambient air, its safe limit was fixed at 8.5 ppm in a European Directive [5].

Nicotine is a specific, sensitive and valid marker of atmospheric SHS that is absent in tobacco smoke-free environments. Air nicotine can be used to estimate respirable particulate matter (PM) exposure from SHS in indoor environments if smoking occurs regularly, if the system is in a near quasi-steady state and if the sampling time is longer than the characteristic times for removal processes [6].

Despite a background concentration of respirable particulate matter (PM) due to cooking and infiltration from outdoors, PM is considered as a surrogate marker of SHS, at least at relatively high levels [2]. PM < 10 μm enter the respiratory tract, but are primarily produced by mechanical processes such as construction activities, road dust resuspension and wind. PM < 2.5 μm (fine particles) originate primarily from combustion sources (such as smoking). Their background value has been estimated to be 3–5 μg/m^3^ in the US and Western Europe, and the limit of air quality is estimated at 10 μg/m^3^ annual mean and 25 μg/m^3^ 24 h mean. Those limits are those when respectively long *versus* short term health effects are expected to appear. For ultrafine particles (<0.1 μm), no recommendation can be provided, as the existing body of epidemiological evidence on the exposure-response relationship is insufficient [7].

Mathematical models were developed and applied to existing statistics in order to predict respirable SHS suspended particles (<3.5 μm) and nicotine in air inside the home from building smoke density, air exchange rate, exposure duration, *etc.*, to convert serum cotinine into urine cotinine, and to estimate atmospheric SHS-PM and nicotine from cotinine in vital products. These models, called “Rosetta Stone” Equations by Repace, permit broad comparison of clinical and epidemiological studies using respectively atmospheric markers and biomarkers for SHS [8]. For each microgram of atmospheric nicotine, there is an estimated increase of about 10 μg in respirable SHS particle concentration [1].

Fluorescing particulate matter (FPM) and ultraviolet-absorbing particulate matter (UVPM) are also sensitive atmospheric markers [1], although not frequently used. The carcinogenic particulate polycyclic aromatic hydrocarbons (PPAH) are also used for measuring air quality [9,10]. Benzene, another carcinogenic substance of tobacco smoke, is sometimes used as an air marker of SHS (UE security limit for ambient air: 5 mcg/m^3^ [5]. For volatile organic compounds and semi-volatile compounds, sorption on and desorption from indoor surfaces influence exposure.

### 2.2. Biomarkers

The characteristics of an ideal SHS biomarker are the following: specificity for tobacco, appropriate half-life, sensitivity and precision, sampling with non-invasive techniques, low cost, association with health effects or with an agent having health effects, quantitative relationship to prior exposure to SHS [1].

*CO* in exhaled air and blood *HbCO* are both inadequate biomarkers of exposure to SHS due to poor specificity and a short half-life. *Thiocyanate* is inadequate due to lack of sensitivity. Nicotine is specific, but its variations due to its 1–3 h half-life limit its value as a marker of exposure. When measured in hair (where it is incorporated into the growing shaft over time), nicotine seems to be a better marker of chronic exposure. Hair nicotine levels were better able to identify infants aged 3–27 months according to smoking in their households (no smoking, smoking only outside the home, smoke inside the home) than urine nicotine (p < 0.0001), and were correlated with the number of smokers [11]. An evident positive association between hair nicotine concentrations in non-smokers and higher numbers of cigarettes smoked per day in a household was demonstrated [12]. Of a total of 1,746 cases from six US, Canada and France databases, the cut-off value of hair nicotine distinguishing active smokers from passive smokers or subjects unexposed to smoke were 0.8 ng/mg for non-pregnant and 0.2 ng/mg for pregnant women. The cut-off value between exposed and unexposed children was 0.2 ng/mg [13].

In 2,485 women participating in the Nurse's Health Study in 1982, toenail nicotine levels differed significantly according to reported smoking status (median level for non-smokers without SHS exposure 0.10 ng/mg; with SHS exposure 0.14 ng/mg; and for active smokers 1.77 ng/mg), but with considerable overlap in nicotine levels between the reported types of smoking status [14]. Its value is thus limited.

Cotinine, the main metabolite of nicotine, is used most frequently as its half-life (±16–18 h) [15] allows levels to remain fairly constant during the day. The cut-off point of 14 ng/mL *plasma cotinine* currently used overestimates the number of non-smokers, and a new overall cut-off point of 3 ng/mL between smokers and non-smokers has been proposed, based on data from 3,078 smokers and 13,078 non-smokers. This shift is due to the decline in SHS exposure in the US in recent years [16].

Optimal end-points for *saliva cotinine* concentration between smokers and non-smokers vary according to the presence (18 ng/mL) or absence (5 ng/mL) of smoking in the home [15]. Saliva cotinine concentration closely parallels serum concentration (saliva cotinine concentration = serum × 1.1 to 1.5) [17–20].

*Urine cotinine* concentrations are also highly correlated with blood concentrations (r ± 0.8) [20–22]. The ratio of urine to plasma cotinine is on average 4 to 5 [23]. Correction of urine cotinine for creatinine concentration improves the correlation between urine and plasma cotinine (R = 0.91–0.95). A plasma cotinine concentration of 1 ng/mL corresponds to a daily intake of 100 μg of nicotine [24].

Inter-subject variability reduces the accuracy of the relationship between nicotine intake and plasma cotinine for individuals in comparison with population studies. Cotinine measures from single urine samples provide accurate estimates of recent exposure (2–3 days) in groups of children, but for estimates of the mean cotinine levels of an individual child over 4–15 days, up to nine urinary samples may be necessary, and up to 12 urinary samples for exposure over a 4- to 13-month period [25].

The different cut-off points distinguishing between cigarette smokers and non-smokers from different racial/ethnic groups (between 1 and 6 ng/mL *vs.* the overall cut-off point of 3 ng/mL serum cotinine [16]) are probably related to racial differences in global levels of exposure in daily life, as after exposure of 40 non-smokers for 4 hours (men and women, African-Americans and whites) to aged diluted sidestream smoke in an environmental chamber under uniform conditions, the increase in biomarker levels (cotinine or (4-methylnitrosaminol)-1-(3-pyridyl)-1-butanol [NNAL] or 4-aminobiphenyl [4AB] adducts) were similar in all groups [26].

The cut-off point between smokers and non-smokers exposed to SHS is also influenced by the fact that in non-daily smokers, who represent more than 30% of cigarette smokers [27], the short-term biomarkers of exposure could decrease to non-smoker levels in the period between smoking episodes.

A 24 h urine collection of the sum of four or six of the major nicotine metabolites is correlated better than cotinine with small doses 100–400 μg (in the range of SHS) of deuterium-labeled nicotine administered per os daily for 5 days [28].

Nicotine is detected by colorimetry and, as well as cotinine, by various immunologic and chromatographic methods, completed or not with mass spectrometry. Immunologic techniques are adequate for large samples and chromatographic methods for more precise and limited studies.

Serum cotinine measurements were compared in experienced laboratories using either gas-liquid chromatography with nitrogen-phosphorus detection or liquid chromatography with mass spectrometry detection in concentrations ranging from 0.05 ng/mL to high concentrations of active smokers. Accurate and precise results were observed without interlaboratory bias [29].

The combination of a biomarker and self-reported exposure is considered a better way of estimating exposure than serum cotinine levels alone, as time delays between SHS exposure and blood collection can influence cotinine values. In the Monica Study, the kappa coefficient for agreement between self-reported exposure (in various questionnaires) and serum cotinine levels is always <0.24, considered as only a fair agreement between the two methods [30]. The level of agreement differs in other studies due to differences in wording of questions, type of population, type of analysis, *etc*. Among 183 pregnant Polish women, for example, serum cotinine levels corresponding to active smoking were detected in 17% of self-reported unexposed non-smokers and levels corresponding to SHS exposure in 74% [31].

Dipstick tests (NicAlert and TobacAlert strips) were used in urine and saliva for qualitative assessment of cotinine levels. In urine samples both TobacAlert and NicAlert performed poorly at low nicotine levels, and cannot be used reliably to indicate a specific level of urine cotinine at the low levels found in SHS-exposed people [32]. The specificity of NicAlert in saliva is 95%, its sensitivity 93%, its positive predictive value 95% and its negative predictive value 93% for verifying the smoking status (>10 ng/mL) of self-identified smokers *versus* non-smokers with gas chromatography-nitrogen phosphorus detection as reference standard. In the readings from all five non-smokers exposed to SHS (among a total of 45 non-smokers), the NicAlert test was always negative. Thus the method did not detect all levels of SHS exposure [33].

Protein and DNA adducts are not often used for the detection of SHS exposure. Due to their half-life of ±40 days, urine NNAL and NNAL glucuronide [NNAL-*O*-gluc] and [NNAL–*n*-gluc] (metabolites of the carcinogenic tobacco specific nitrosamine NNK [4-(methylnitrosaminol)-1- (3-pyridyl)-1-butanone] can monitor long periods of exposure and are well correlated with urine cotinine levels. In a review of the literature, NNAL and NNAL-glucuronide were the most consistently elevated biomarkers in people exposed to SHS [34].

In urine samples, the levels of 1-hydroxypyrene-*O*-glucuronide, the major metabolite of pyrene, a common polycyclic hydrocarbon, correlated (p = 0.04) with the hours of secondhand exposure in non-smokers; poor specificity, however, does not allow them to be used to measure SHS exposure [35]. Other carcinogenic products, benzene and 1-3-butadiene, were also measured in air and in body fluids of non-smokers [36]

Biomarkers can allow a valuable quantification of tobacco smoke absorption but can also establish the biological plausibility of the epidemiological observations [37].

## 3. Exposure to SHS

Exposure to SHS is indirectly evaluated through studies of air markers or can be measured directly by biomarkers of intake. Both can aid in selection of preventive measures (e.g., partial or total smoking bans) and assess their efficiency and implementation.

### 3.1. Air Markers

#### 3.1.1. Hospitality venues

Before the smoking bans in hospitality venues high concentrations of SHS were demonstrated mostly by large PM_2.5_ levels of above the American safety standard of 35 μg/m^3^ per 24 h [38]. This was the case for pubs, cafes and bars [39], particularly in pubs frequented by deprived communities *versus* affluent communities [40], but also and even at higher levels in discotheques and at lower levels in restaurants and restaurant-cars [39].

That ventilation fails to control SHS was demonstrated by the high levels of PM_2.5_ and PPHA in pubs despite a per occupant ventilation rate that conformed to the recommendations [10].

Even in outdoor dining areas, but at only one meter of a smoker, the PM_2.5_ levels, although below the safety standard, triple during the smoking period. Being under an overhead awning increases the average exposure to PM_2.5_ by around 50% in comparison with the background level [41].

The same trends were observed when air nicotine (lower limit of detection 0.01 μg/mL) was detected in a cross-sectional study of hospitality venues in eight European countries: nicotine was detected in 97.5% of the samples. The highest median values were observed in discos/pubs, and the lowest in restaurants. The concentrations were higher in smoking *vs.* non-smoking areas (ratio 3.12). Most countries had only smoking restrictions or no regulations at all, but in Ireland and Italy, where a smoking ban had been implemented, the nicotine concentrations were much lower [42]. There was a trend towards lower concentrations in this 2008 study in comparison with the earlier 2005 data in hospitality venues [43].

In capital cities of Latin America, the highest levels of nicotine were measured in bars, but a median concentration of 0.60 μg/m^3^ nicotine was also detected in the non-smoking areas of restaurants, showing that unisolated non-smoking areas are not fully effective in controlling SHS exposure [44].

PM_2.5_ concentrations were compared in 32 countries from the five regions of the World Health Organization (WHO). The levels were 7.5 times higher in countries without indoor air policies compared with countries with such policies. The concentrations were also much higher in venues where smoking was noticed than in those where it was not; they are linked with the average number of cigarettes (or water-pipes) per 100 m^2^ [45]. In venues where smoking was noticed, the observed levels were well above the WHO quality targets of 10 μg/m^3^ annual mean and 25 μg/m^3^ 24h mean [7].

#### 3.1.2. Hospitals

Air nicotine concentrations were quite dissimilar in the hospitals of five European cities in 2001–2002 (ranging from 0.14 to 4.0 μg/m^3^) [43]. In Latin America, despite restrictions or bans on smoking, low levels of nicotine were detected in 95% of hospital samples, with a large range within and across hospitals (0.01–1.33 μg/m^3^) [44].

#### 3.1.3. Aircrafts, airports and train stations

In-flight air quality measured in ±250 American aircrafts before the 1989 smoking ban violated the PM_2.5_ federal air quality standards in the cabins by approximately three fold for flight attendants due to major deficiencies in the ventilation systems [46]. In seven European cities in 2001–2002, despite smoking restrictions in most areas, ranges of air nicotine concentrations were 0.1–5 μg/m^3^ in airports and 0.5–10 μg/m^3^ in train stations [43].

#### 3.1.4. Motor vehicles

In smokers’ vehicles, median air nicotine concentrations were 9.6 μg/m^3^, whereas there were non-detectable concentrations in non-smokers’ vehicles. The increase was 1.96 fold per cigarette smoked. The level of air nicotine was higher than in restaurants and bars [47]. In 18 motionless cars, windows closed, the average levels of PM_2.5_ were >3,800 μg/m^3^; with air conditioning, the levels decreased to 844 μg/m^3^, and holding the cigarette next to a half-open window, to 223 μg/m^3^ [48].

In three stationary cars in Crete, where the outside baseline measurements were 13 μg/m^3^, with a cigarette left to burn until it extinguished itself, and without heating/air conditioning, the mean PM_2.5_ was between 12,000 and 13,000 μg/m^3^; it decreases significantly with increase in air circulation (from windows fully closed, to half open or fully open), and in vehicles with a large interior passenger volume [49]. The nicotine level in air sample, surface wipe and dust from used cars for sale was higher in 87 smokers’ cars than in 20 non-smokers’ cars. When smokers had imposed a car smoking ban, the air nicotine levels were significantly lower, but dust and surface contamination levels remained at similar levels. This shows the importance of the issue of “third hand smoke” [50].

#### 3.1.5. Various public places

In a review, the weighted means of indoor air nicotine obtained from 13 studies conducted in the US before 2003 were as follows: 3 betting establishments: 9.8 μg/m^3^; 2 bowling alleys: 10.5 μg/m^3^; 2 billiard halls: 13.0 μg/m^3^; 10 bars 31.1 μg/m^3^; 2 bingo parlors: 76.0 μg/m^3^ [51]. In China, a mean nicotine level of 7.48 μg/m^3^ was observed in entertainment establishments *versus* 2.17 μg/m^3^ in restaurants [52].

#### 3.1.6. Schools and universities

In seven European cities, median air nicotine concentration ranged from 0.07 to 0.80 μg/m^3^ in schools and from 0.01 to 0.50 μg/m^3^ in universities, with similar data whether or not smoking was allowed in a particular area [43]. In Latin America, despite a smoking ban in most countries, nicotine was detected in 78% of samples, but at a low median concentration [44].

#### 3.1.7. Homes and residences

Since the implementation of bans of smoking in public places, homes have become a major contributor to SHS exposure.

PM_2.5_ was measured over a 3-day period in nine homes where smoking was allowed and in three smoke-free homes [53]. PM_2.5_ levels in the primary smoking area of the smoking homes were significantly higher than in distal areas (84 *vs.* 63 μg/m^3^). Smoking in only part of the home offers thus no complete protection for people anywhere inside the home. In smoke-free homes, PM_2.5_ levels were 9 μg/m^3^ lower than the Environmental Protection Agency (EPA) annual standard for air quality (15 μg/m^3^).

A partial explanation of the persistence of SHS in homes where a smoker is living but not smoking inside the home could be the washout time for residual tobacco smoke from the lungs after the smoker’s last puff, as 10 repeated re-entries of a smoker after the last puff increases the PM_2.5_ in the room from a background of 0.56 μg/m^3^ to 3.32 μg/m^3^ [54].

In the Greater Boston Area, air nicotine concentrations and the air exchange rates were measured in 49 low-income multi-unit residences. There was a wide range of levels, from the limit of detection to 26,92 μg/m^3^, with a mean value of 2.20 μg/m^3^. Smoke contamination is not limited to houses with smokers, but increased levels of nicotine concentration were observed in non-smoking homes, suggesting SHS infiltration from neighboring units [55]. PM_2.5_ levels measured through personal monitors worn by 407 non-smoking pregnant Polish women were higher when exposed to >10 cigarettes per day (CPD) (88.8 μg/m^3^) than when exposed to ≤10 CPD (46.3 μg/m^3^) or unexposed to SHS (33.9 μg/m^3^) [56].

#### 3.1.8. Water-pipe smoking

During laboratory sessions, the levels of PM_2.5_ in the air increased from 48 μg/m^3^ at background to 264 μg/m^3^ after water-pipe smoking by 10 individuals, while after smoking 10 cigarettes the levels increased from 44 μg/m^3^ to 267 μg/m^3^. Water-pipe smoking thus contributes as much to SHS as cigarette smoking [57].

### 3.2. Biomarkers

#### 3.2.1. General population

A decrease in SHS exposure has been observed in the USA [58,59]. In the cross-sectional National Health and Nutrition Examination Survey (NHANES) surveys of the US civilian population aged ≥4 years, among non-smokers (serum cotinine ≤ 10 ng/mL), exposure to SHS (defined as a detectable *serum cotinine* level of ≥0.05 ng/mL) declined significantly from 83.9% in 1988–1994 to 46.4% in 1999–2004. The percentage of decline was lower among the younger population aged 4–19 years than for those aged ≥20 years, and smaller for the lowest income group compared with the higher income groups. This decrease can be linked with the smoking bans and the reduced prevalence of active smoking [58].

The prevalence of *serum cotinine* levels ≥0.05 ng/mL in the non-smoking US population decreased significantly, from 52.5% during 1999–2000 to 40.1% during 2007–2008. Nevertheless, an estimated 88 million non-smokers aged ≥3 years are still exposed. Children remain among the most exposed [59]. Among a nationally representative sample of 4,952 non-smoking employed adults who reported no home exposure to cigarette smoke, the geometric mean *serum cotinine* (ng/mL) ranged from 0.09 for farming, foresting and fishing occupations to 0.22 for operators, factory workers, laborers and to 0.47, the highest, among waiters for the period 1988–1994, with values decreasing between 1988 and 1994 [60].

In US, with a cut-off point of ≥0.05 ng/mL *serum cotinine* for adult non-smokers, the prevalence rates of exposure to SHS in the general population were 44.9% nationally (4,476 subjects in the 2003–2004 survey) and 56.7% in New York City (2004 survey), respectively, contrasting with active smoking prevalences of 29.7% and 23.3%, respectively [61].

Among African-American or Dominican pairs of women and newborns residing in underserved neighborhoods of New York City with levels of plasma cotinine <25 ng/mL, 53% of mothers and 49% of infants had levels indicative of SHS exposure (≥0.03–<25 ng/mL) [62].

#### 3.2.2. Hospitality venues

Twenty four h urine samples were collected on working and non-working days in non-smokers working in restaurants and bars that permitted smoking. Significant increases in *nicotine + nicotine-glucuronides*, *cotinine + cotinine N glucuronide* and *total NNAL* were observed during the working period [63].

In New Zealand hospitality venues, the saliva cotinine concentrations increased more between the beginning and the end of the workshift among workers in premises allowing smoking among customers everywhere than in smoke-free premises, and in premises permitting smoking only in designated areas [64].

In Dakota, USA, after exposure to SHS in three heavily polluted bars, the net mean increases in *urinary cotinine* among eight healthy non-smoking patrons were measured before and estimated after 6 hours (from the mean of 2 h and 12 h measurements). The post-exposure values in the three bars were 4.28 ng/mL, 6.88 ng/mL and 9.55 ng/mL, respectively, above the pre-exposure background values [65].

Prior to the smoking ban, the increase in *saliva cotinine* was measured after 3-hour exposure among non-smokers in bars in New Zealand: the mean increase was 0.66 ng/mL, *i.e.*, ±8-fold, higher in winter than in spring, even in venues that seemed to be smoke-free on direct observation [66].

In Hong Kong, among 104 workers in catering facilities not exposed outside the workplace, mean *urinary cotinine* levels ranged from 6.4 and 6.1 ng/mL when smoking was not allowed or restricted to 15.9 ng/mL with unrestricted smoking [67].

*Urine concentrations of cotinine* were measured in 100 non-smoking volunteers before entering a Mexican discotheque and 6 hours after the end of the exposure. In males, pre-exposure levels of cotinine (3.7 ng/mL) increased to 49.1 ng/mL post-exposure and in females from 2.2 ng/mL to 15.7 ng/mL [68].

A volunteer sample of bar (86%) and restaurant (14%) workers was recruited for a *hair nicotine* prevalence study. SHS exposure at work, at home, in the car or truck and in any other source of regular exposure was self-reported. Among 129 non-smokers, the hair nicotine levels were 0.63 ng/mg with no exposure, 1.18 ng/mg with one source, 1.32 ng/mg with two and 1.96 ng/mg with three or more; type of establishment, bar *versus* restaurant (p = 0.001) and number of sources (p = 0.007) were the best predictors of hair nicotine level. The total variance explained by the model is only 12%. The total hours of reported exposure were associated with both the number of sources (p < 0.0001) and hair nicotine level (p < 0.0001) [69].

#### 3.2.3. Casino patrons

Mean increases in *total* urine *cotinine* (0.044 nmol/mg creatinine) and *total NNAL* (0.018 pmol/mg creatinine) were significant (p < 0.001) among non-smoker casino patrons before and after 4 hours stay in a casino where smoking was allowed [70].

#### 3.2.4. Hospital inpatients

In a public hospital of San Francisco, in 2005–2006, significant exposure to SHS (possibly before hospitalization) was demonstrated through measurement of *serum cotinine* levels in 32% of inpatients denying smoking [71].

#### 3.2.5. Aircraft

In flight attendants, before the smoking ban in aircrafts, SHS assessed through *cotinine urinary* dosimetry was estimated to be 14-fold that of the average person. Ventilation declined by 33–60% between 1970 and 1987, and massively failed to control SHS air pollution in aircraft cabins [46].

#### 3.2.6. Home

In the Health Survey for England, nationally representative samples of children aged 4–15 years (n = 13,365) and their parents were interviewed in the home. The proportion of homes where one parent was a smoker but that were smoke-free, increased from 21% in 1996 to 37% in 2007, and where both parents smoked but were also smoke-free, from 6% to 21%. Most homes with non-smoking parents were smoke-free (95% to 98%). The geometric mean *saliva cotinine* across the years was 0.22 ng/mL for children with non-smoking parents living in a non-smoking home. When one parent was smoking, the mean cotinine levels were 0.37 ng/mL in non-smoking homes and 1.67 where there was smoking in the home. For the children with two smoking parents, these values were 0.71 and 2.46 ng/mL, respectively. Across the years, there was a declining trend in saliva cotinine concentration in smoke-free homes for children of smoker or non-smoker parents [72].

In Poland, one year after delivery, 46% of children are exposed to SHS as confirmed by *urine cotinine* measurements [73].

In Spain, in a cohort study of 987 infants, the median *urinary cotinine* increases 1.4 times when father smokes and 3.5 times when mother smokes. In most children considered unexposed to SHS by their mother, *urine cotinine* levels above 1 ng/mL are detected [74].

*Hair cotinine* was measured in 104 mother/child pairs. In general, child hair cotinine levels were higher than maternal levels (1.18 ng/mg *versus* 0.78 ng/mg, p < 0.001). Levels in children of nonsmokers were higher than maternal levels (0.77 ng/mg *versus* 35 ng/mg, p < 0.001) while levels were not different between smokers and their children (1.91 ng/mg *versus* 1.92 ng/mg, p = 0.978) [75]. Geometric means of *hair nicotine* of pregnant women are the lowest (0.33 ng/mg) when the spouse is not smoking (A), intermediate (0.51 ng/mg) when the spouse is a smoker not smoking in the home (B) and 0.58 ng/mg when the spouse is smoking in the home (C). Differences between A and B and A and C are significant, but not between B and C. Not smoking only in the home is thus inadequate to protect completely pregnant women from SHS [76].

Pregnant women frequently underestimate their exposure to SHS. In a study of 698 pregnant women reported as non-smokers, 305 (43.7%) claimed not to be exposed to SHS, yet 196 of these (64.3%) had *plasma cotinine* levels above the limit of detection [77].

Among 114 infants living in homes with parents who smoked, *urine total NNAL* was detectable in 67, for whom the number of cigarettes smoked per week in the home or car was significantly higher. The mean level of NNAL in the 144 infants was 0.083 pmol/mL, higher than in most other field studies of SHS, probably linked to the proximity of infants to parents who smoke [78].

In families where the pregnant women was neither smoking nor exposed to external sources of SHS, and where the father was smoking indoors, the *hair nicotine* concentrations of the neonate are significantly higher (p < 0.05) than in the group with non-smoking fathers or fathers smoking only outdoors. Paternal smoking inside the home thus produces maternal and fetal intake of SHS [79]. Children’s *urine cotinine* decreased non-significantly in children of mothers receiving counseling to reduce children’s secondhand exposure *versus* a control group of mothers with usual care [80].

#### 3.2.7. Experimental conditions

Forty non-smokers were exposed for 4 hours to aged, diluted SHS generated by a smoking machine (140 μg nicotine/m^3^). Short-term increases in *saliva cotinine* were similar, at approximately 12 pg/mL/min, among men and women, African-Americans and Whites, suggesting an absence of metabolic differences between the groups [26].

#### 3.2.8. Pets

*Urinary cotinine* was detected in 15 domestic dogs from homes where residents smoked at least 20 CPD for a minimum of 24 months, and not in 15 dogs from homes without smokers. In exposed dogs, broncho-alveolar lavage detected an increase in macrophage and lymphocyte population [81].

Cats from 19 households in which smoking was reported had significantly higher concentrations of *urine total nicotine* (70.4 ng/mL), *total cotinine* (8.53 ng/mL) and *total NNAL* (0.0562 pmol/mL) compared with cats that lived in 42 households in which there was no smoking. (4.89 ng/mL, 0.74 ng/mL and 0.0182 pmol/mL, respectively [82]. The risks incurred by their pets can sometimes push smokers towards cessation.

## 4. Effects of Smoking Bans

Air and biomarkers measurements allow both the effectiveness of the smoking bans and the level of their effective implementation to be quantified.

### 4.1. Effects on Validated Air Quality

#### 4.1.1. Hospitality venues

Major improvements were observed in hospitality venues of many countries after the introduction of smoking bans. The levels of *PM_2.5_* were measured before and after the 2003 New York State law on smoke-free workplaces in a purposeful sample of 20 hospitality venues. After the ban, a general decrease of 84% of PM_2.5_ was observed. In bars and restaurants where smoking was occurring at baseline, a decrease of 96% was observed (towards mean values below the security standard). In the restaurant portion of the two bar-restaurants initially allowing smoking in the bar but not in the restaurant section, the decrease averaged 58% in the restaurant section. In four large recreation venues (two bowling alleys, a pool hall and a bingo hall) there was a 76% decrease [83].

In Wilmington, Delaware, where the smoking ban was extended in 2002 to hospitality venues, the air *PM_3.5_* and *PPAH* were measured in a casino, six bars and a pool hall/bar where smoking was initially allowed. The average prelaw values of PM_3.5_ were 231 μg/m^3^ *vs.* 9.5 μg/m^3^ outdoors and 134 ng/m^3^ PPAH *vs.* 21 ng/m^3^ outdoors. Mean post-ban values are indistinguishable from outdoors. Ventilation or air cleaning would be unable to obtain such a decrease [9].

After the smoke-free workplace law in Boston, MA, the *PM_3.5_* decreased by 23-fold (to an average of 7.7 μg/m^3^) in six pubs, and the *PPAH* by nearly 10 times, to 6.32 ng/m^3^. PM reached the levels of good air quality in 6/7 venues, and PPAH levels were lower than outdoors in the seven pubs [10].

The effects of the Irish smoking ban in bars, restaurants, cafes and hotels (excluding bedrooms, outdoor areas and properly designed smoking shelters) were assessed via *air nicotine* measurements. There was an 83% reduction in air nicotine concentration from a median 35.5 μg/m^3^ to 5.95 μg/m^3^ (p < 0.001). Validated exposure and the number of observed hours of exposure were thus reduced but not totally eliminated after this partial ban [84]. In 42 Irish pubs, a *PM_2.5_* reduction of 83% was observed after the ban. In 26 pubs, *benzene* concentrations were reduced by 80.2% [85].

In the English hospitality industry, indoor *PM_2.5_* concentrations decreased by 95% from 217 μg/m^3^ at baseline to 11 μg/m^3^ one month after the ban [86]. In a random selection of 41 pubs in two Scottish cities, the levels of *PM_2.5_* averaged 246 μg/m^3^ before and 20 μg/m^3^ after the ban. In most pubs, the level of PM_2.5_ became comparable with the outside ambient air PM_2.5_ level. The levels of *benzene* decreased also from 18.8 μg/m^3^ preban (above the security limit of 5 μg/m^3^) to 3.6 μg/m^3^ postban. Reductions were observed in all post-ban visits, showing high compliance with the ban [87].

Full shift *PM_2.5_* geometric means were measured with personal PM_2.5_ monitors in six bar workers in Scotland. They fell from 202 μg/m^3^ pre-ban to 28 μg/m^3^ post-ban, meaning (after log analysis) an average reduction of 86% [88].

In selected restaurants and bars with a serving area larger than 100 m^2^ in Finnish cities, measurements were conducted before and after enforcement of the act imposing smoke-free zones in such venues. The overall *air nicotine* concentrations decreased in 10 of 18 establishments. The geometric mean nicotine concentration moved in restaurants from 0.7 μg/m^3^ to 0.6 μg/m^3^, in bars and taverns from 10.6 μg/m^3^ to 12.7 μg/m^3^, in discos and nightclubs from 15.2 μg/m^3^ to 8.1 μg/m^3^. In the smoke-free sections, the concentrations were practically unchanged (2.9 μg/m^3^). The small improvements in some sites were linked with increased ventilation, but physical barriers separating smoking from smoke-free areas are thus required to obtain a major reduction in SHS [89].

In 13 Norwegian bars and restaurants, the arithmetic mean concentration of *air nicotine* declined from 28.3 μg/m^3^ to 0.6 μg/m^3^ after the total smoking ban. *Total dust* declined from 262 μg/m^3^ to 77 μg/m^3^ [90].

In nine Swedish communities (bingo halls, casinos, bars and restaurants), exposure above the *air nicotine* cut-off level chosen to identify possible health risk (<0.5 μg/m^3^) existed in 87% before and 22% after the smoking ban; the pre-ban exposure levels were highest in bingo halls and casinos [91].

In Australia, where an indoor smoking ban was promulgated, in a convenience sample of pubs and bars that had at least one indoor area with an adjacent semi-enclosed eating area, *PM_2.5_* concentrations were reduced indoors by 65% (p = 0.004) and outdoors by 38.8% (p = 0.037) after the ban. However, after adjustment, a 100% post-ban increase in geometric mean outdoor PM_2.5_ was associated with a 36.1% increase in geometric indoor PM_2.5_, showing that the air quality in smoke-free indoor areas may be compromised by smoking in adjacent outdoor areas [92].

The indoor air quality was compared by measuring *PM_2.5_* before and after enforcement of a smoking ban in Italy in 40 public places (bars, pubs, restaurants and game rooms). PM_2.5_ decreased from 119.3 μg/m^3^ to 38.2 μg/m^3^ after 3 months (p < 0.005) and to 43.3 μg/m^3^ one year later. The mean outdoor measurements of PM_2.5_ were 20.8 three months post-law and 27.2 one year post-law [93].

#### 4.1.2. Prisons

In 22 dormitory and common areas in six North Carolina prisons, the *PM_2.5_* levels decreased by on average 77% after the indoor smoking ban [94]. This is particularly important, as the prevalence of tobacco use is much higher among inmates and prison staff than in the general population.

#### 4.1.3. Public places, industrial service sector and office workplaces

In eight Finnish public and private workplaces, median indoor *airborne nicotine* concentrations decreased from 0.9 μg/m^3^ before to 0.1 μg/m^3^ one year after and then remained unchanged 3 years after implementation of the national smoke-free legislation [95].

In Montevideo, Uruguay, a middle income country, the median *air nicotine* concentrations of 100–103 indoor samples were 0.75 μg/m^3^ before compared to 0.07 μg/m^3^ after the legislation, but with a large overlap of the interquartile rate (IQR) between the two periods. The overall reduction was 91% after adjustment for room volume and ventilation. The greatest reduction was observed in schools (97%), followed by airports (94%), hospitals (89%), local government buildings (86%) and restaurants/bars (81%). The reason for the much lower overall air nicotine concentration in Uruguay *versus* other countries is not mentioned, but could be due to the selection process or the differences in prevalence of active smoking or increased ventilation in warm countries and the different patterns of active smoking [96].

#### 4.1.4. Contrast between countries with and without smoking bans

The air quality differences of hospitality premises between a country with (Italy) and without (Austria) a smoking ban was assessed before and 2 years after the introduction of the Italian smoking ban in respectively 28 and 19 hospitality premises. Median *airborne nicotine* concentrations decreased from 8.86 μg/m^3^ to 0.01 μg/m^3^ in Italy (p < 0.001) and, in contrast, increased from 11.00 μg/m^3^ to 15.76 μg/m^3^ in Austria, a non-significant increase [97].

### 4.2. Effects of Smoking Bans on Biomarkers of SHS

Effectiveness and implementation of smoking bans can be measured through direct comparison of biomarkers pre- and post ban in the same areas, and can also be estimated through indirect simultaneous comparisons of biomarkers in areas with and without bans (with possible bias linked with other inter-area differences).

#### 4.2.1. General population

##### 4.2.1.1. Pre- and post comparisons

From October 1988 through December 2002, in a sample of 29,849 individuals representative of the US non-institutionalized civilian population ≥4 years of age, a substantial decline of approximately 70% in *serum cotinine* concentration was observed in non users of tobacco. Serum cotinine concentrations ≥ 0.05 ng/mL were observed in 88% of non-smokers between 1988 and 1991, and in only 43% in 2000–2002. Increase in smoke-free policies and to a small degree reductions in smoking prevalence may have contributed to the decline of the exposure of non-smokers to SHS.

The fact that smoke-free bans are not “applicable” to home and car exposure could explain the higher serum concentration in children, while higher SHS exposure also seems responsible in non-Hispanic blacks [58].

In the general population of New York, *saliva cotinine* levels decreased by 47.5% among non-smoking adults after implementation of the 2003 New York state ban on smoking in indoor workplaces and public places [98].

##### 4.2.1.2. Comparisons between areas

In the US, SHS exposure, defined as *serum cotinine* ≥ 0.05 ng/mL, was determined from 5,866 non smoking adults categorized by their level of coverage by a smoke-free law. The odds of exposure was much lower with extensive coverage (0.10 in men and 0.19 in women) and lower with limited coverage (0.57 in men and 0.90 in women) than with no coverage, used as reference value [99].

#### 4.2.2. Hospitality workers

##### 4.2.2.1. Pre- and post comparisons

In a pre-post follow-up study, *saliva cotinine* specimens were available among 24 non-smoking workers in restaurants, bars and bowling facilities followed up until 12 months after the New York smoke-free law. Saliva cotinine concentration decreased from 3.6 ng/mL (95% confidence interval [CI] 2.6–4.7) to 0.8 ng/mL (95% CI 0.4–1.2), suggesting compliance with the law [100].

The effects of the Lexington smoke-free law covering all buildings open to the public were investigated respectively before and 3 months after the law was implemented in a convenience sample of 105 workers (including 40 active smokers), of whom 92 worked in restaurants and 13 in bars. The geometric mean of *hair nicotine* among bar-workers was 4.51 ng/mg before and 1.06 ng/mg 3 months post-law, and in the restaurant cohort 1.54 ng/mg before and 1.20 ng/mg after. After adjustment for CPD, the decline from pre- to post-law was significant for bar workers (p = 0.0004), but marginal among restaurant workers (p = 0.07). Subject retention was a problem, as only 71 of the 105 initial participants were followed up at 3 months [101].

In Minnesota communities, among 24 non-smokers working in hospitality venues (12 bars, 6 restaurants, 5 bowling alleys and one unknown workplace) and living in non-smoking households, the median percentage decrease after the ban was very significant *in urine* as well for *total cotinine* (83.3%) as for *total NNAL* (76.6%). Levels of *total NNAL/mg creatinine* were significantly correlated with the number of hours worked in the smoking section (r = 0.43; p < 0.05) [102].

In England, among 75 non-smoking employees of the hospitality industry, *saliva cotinine* was reduced from 3.6 ng/mL one month before the smoke-free legislation to 0.9 ng/mL one month after its implementation (p < 0.001) [86].

The same trend was observed in Norway among 25 non-snuffing non-smoker employees in bars and restaurants from a geometric mean *urine cotinine* concentration of 9.5 μg/g creatinine preban to 1.4 μg/g creatinine post-ban (p < 0.01). However, there was also a reduction among 29 non-snuffing smokers, from 1,444 μg/g creatinine to 688 μg/g creatinine (p < 0.05), suggesting a reduction in active smoking after the intervention [90].

The impact of the Irish smoking ban on the *saliva cotinine* concentration in a cohort of 35 workers from 15 city hotels translated into a 69% decrease from a median value of 1.6 ng/mL to 0.5 ng/mL (p < 0.0005), showing the persistence of limited exposure. Self-reported exposure to SHS nevertheless decreased from a median of 30 hours a week to zero [84].

In Italy, the concentration of *urine cotinine* among non-smoker workers in 40 hospitality venues decreased from 17.8 ng/mL to 5.5 ng/mL 3 months after (p < 0.0001) and to 3.7 ng/mL 12 months after (p < 0.0001) the smoking ban, together with a contemporaneous reduction in indoor fine and ultrafine particles [93].

##### 4.2.2.2. Inter-area comparisons

The effects of the New York clean indoor legislation appear also in a pre-postlaw comparison of non-smoking hospitality workers compared with American Indian casino workers exempt from the law. The median *urine cotinine* decreased from 4.93 ng/mL prelaw to 0.30 post-law (p < 0.01) among the former, but remained at a higher level in the latter (8.40 *vs.* 6.49). Urine cotinine levels were significantly related with self-reported numbers of exposures and number of days exposed [103].

In Oregon, the influence of exposure was assessed among CO-validated non-smokers employed in 52 bars or restaurants where smoking was allowed or in 32 where smoking was prohibited by local ordinances. After a workshift of 4 hours, among workers exposed to workplace SHS, the odds of having a detectable *urine level of total NNAL* were almost six times higher than for protected workers (aOR = 5.66; p = 0.005). The same was true for detectable levels of *urine nicotine* (aOR = 109.01; p < 0.001) *and cotinine* (aOR = 95.21; p < 0.001). The post workshift mean level of urine total NNAL, nicotine and cotinine was 0.02 pmol/mL, 1.39 ng/mL and 1.4 ng/mL for protected workers *vs.* 0.04 pmol/mL, 44.36 ng/mL and 20.20 ng/mL for exposed workers. Each hour of self-reported exposure to SHS was associated with an increase of 6% in total NNAL, of 33% in total nicotine and of 12% in total cotinine [104].

Following the smoke-free Spanish law of 2006, in bars and restaurants >100 m^2^, the proprietors were still allowed to provide a separate, independently ventilated smoking area comprising less than 30% of the total floor area. For venues with a floor area <100 m^2^, the owner could choose between a totally non smoke-free or a totally smoke-free venue (in fact only 10–20% of those venues were smoke-free). In Portugal and Andorra, no smoking ban was in effect at the time of the study. In a convenience sample in Spain, Portugal and Andorra, hospitality venues were recruited based on their size (< or >100 m^2^) and area: these venues were pubs, bars, restaurants and discos; 117 non-smoking workers completed the study in Spain and 20 in Portugal and Andorra. Non-smokers were defined as self-reported never or former smokers, with a saliva cotinine concentration <20 ng/mL, a high cut-off level justified by the potentially high exposure to SHS in hospitality venues. Before the Spanish law, the median *stimulated saliva cotinine* concentrations among non-smokers were 2.0 ng/mL in Spain and 1.2 ng/mL in Portugal and Andorra (p < 0.01). In Spain, in venues where smoking was totally banned, the median saliva cotinine decreased from 1.6 ng/mL before the ban to 0.5 ng/mL after the ban (p < 0.01), a 56.6% reduction; in 22 venues where smoking was permitted in designated areas, from 1.8 ng/mL to 1.1 ng/mL (p = 0.0618), a non-significant 31.9% reduction; in 63 venues where smoking was permitted throughout the premises, saliva cotinine remained stable from 2.5 ng/mL to 2.6 ng/mL (p = 0.475). In Portugal and Andorra, the median saliva cotinine remained unchanged before (1.2 ng/mL) and after (1.2 ng/mL) the Spanish ban. Self-reported duration of SHS exposure decreased by 100% in Spanish venues where smoking was totally banned (p < 0.01), whereas a borderline significant decrease (from 8 hours median exposure per day to 1 hour per day; p = 0.055) was observed in venues where smoking was partially permitted. Median duration of exposure increased significantly in venues with no smoking restrictions (from 8.0 to 10.0; p < 0.01).

In Portugal and Andorra, duration of exposure to SHS in hospitality venues remained unchanged, at 8 hours. SHS exposure outside the workplace decreased in Spain regardless of the type of postban regulation (between −16% and −54.6%) but increased in Portugal and Andorra (+54.2%) [105].

#### 4.2.3. Bar workers

##### 4.2.3.1. Pre-and post comparisons

In 73 non smoking barmen working in Dublin pubs, there was a 79% reduction in *exhaled breath CO* and a 81% reduction in *saliva cotinine* after a total workplace smoking ban, together with a 90% decrease in total exposure at work, and a 42% decrease outside of work [85].

The same type of comparison was conducted in Canada between Toronto (79 eligible non-smoking bar workers), where a smoke-free bylaw was enacted and Windsor (49 eligible non-smoking bar workers), with no bylaw change. *Urinary cotinine* levels decreased from 24.2 ng/mL to 7.8 ng/mL in Toronto immediately after the ban, with the reduction sustained 1, 2 and 9 months post-ban, while there was no change among Windsor bar workers. The compliance with the ban was both high and persistent [106].

Prior to the introduction of the Scottish smoke-free legislation and 2 months and 1 year after its implementation, saliva samples were collected in their places of work in 191 bar workers. In non-smokers, the *saliva cotinine* level decreased from 2.94 ng/mL to 0.41 ng/mL at 1 year (a reduction of 89% in non-smokers’ cotinine levels). Among smokers, a reduction of 12% was recorded during the same period, probably reflecting both reduction of exposure to SHS at work and a decrease in active smoking [88].

The same trend was observed for *benzene* and *1-3 butadiene* levels, for which the average dose after a standard exposure of 3 hours in Irish pubs diminished by 91 and 95%, respectively, after the ban [36].

##### 4.2.3.2. Inter-area comparisons

In the Republic of Ireland, among 158 non-smoker bar staff, a decrease in *saliva nicotine* concentration was observed before and after legislation for smoke-free workplaces (from 29.0 nmol/L to 5.1 nmol/L), while in Northern Ireland, without a ban, over the same period the decrease was much more limited (25.3 nmol/L to 20.4 nmol/L) and not significant, with overlapping of the confidence intervals. After adjustment, the decline was twice as great in the Republic than in Northern Ireland [107].

#### 4.2.4. Bar patrons

In Dakota, USA, in three heavily polluted bars, the net mean increases in *urine cotinine* among eight healthy non-smoking volunteers were measured before and estimated after a 6-hour exposure (from the mean of 2 h and 12 h measurements). The post-exposure values in the three bars were 4.28 ng/mL, 6.88 ng/mL and 9.55 ng/mL above the pre-exposure background values [65]

*Saliva cotinine* levels were measured among non-smoking volunteers before and after a 3-hour visit to 30 randomly selected bars in New Zealand. Before the comprehensive smoke-free legislation, there was an average increase in saliva cotinine of 0.66 ng/mL. After the law change, the mean saliva cotinine increase was much lower, but persisted at 0.08 ng/mL, despite quasi total compliance with the law (only 1 lit cigarette observed in 30 visits) [108].

#### 4.2.5. Homes

Since the smoking bans in many countries, the home has become a major source of exposure to SHS. In Scottish adult non-smokers from non-smoking households, geometric mean *saliva cotinine* concentrations fell by 49% (from 0.35 ng/mL to 0.18 ng/mL, p < 0.001) within 1 year after implementation of the smoke-free legislation in enclosed public places and workplaces. A smaller (16%) fall in cotinine concentration in non-smokers from smoking households was not statistically significant. The reduction in self-reported exposure to SHS was clear in public places, but not in homes and cars [109].

#### 4.2.6. Adolescents and children

In a national cross-sectional survey of the US population, *serum cotinine* levels were available for 11,486 non-smoking children and adolescents (3–19 years) from 117 survey locations with extensive *versus* no coverage by a smoke-free law. Among those without home SHS exposure, in a county with extensive coverage of a smoke-free air law, the adjusted prevalence of detectable cotinine was 0.61 times lower and the geometric mean 0.57 times lower compared with children and adolescents from counties without a smoke-free air law. In children and adolescents with home SHS exposure, after adjustment for covariables, the differences between counties with or without smoke-free air laws was no longer observed [110].

In a nationally representative cross-sectional survey of ±2,500 children in Scotland, the geometric mean *saliva cotinine* concentrations in non-smoking children fell from 0.36 ng/mL to 0.22 ng/mL after the introduction of the smoke-free legislation (a 39% reduction). The extent of the fall was statistically significant only among pupils living in households in which neither parent figure smoked (from 0.14 ng/mL to 0.07 ng/mL) and among pupils in households in which only the father figure smoked (from 0.57 ng/mL to 0.32 ng/mL) [111].

In Australia, feedback to parents of asthmatic children of the results of the *urinary cotinine to creatinine level* of their children did not significantly change the prevalence of smoking bans at home or in the car, nor the daily consumption of cigarettes or consumption in front of the child [112].

#### 5. Health Effects of Exposure to SHS

The health effects of exposure to SHS concern the general population, hospitality workers (for whom exemptions from the smoking bans are frequently considered), non hospitality workers and finally children (as smoking bans do not concern private housing). The types of effects are disease symptoms, disease prevalence and issues, hospitalization rates for SHS exposure related diseases and biological data possibly related to exposure-related diseases.

As the reader’s interest is frequently disease-oriented, the main exposure-related diseases, *i.e.*, cardiovascular, respiratory diseases in adults and children, fertility and pregnancy, sudden infant death syndrome, cancers, neuropsychiatric diseases and genotoxicity, will be considered successively.

### 5.1. Cardiovascular Diseases

In 20.8% of a nationally representative sample of 13,443 English and Scottish adults, high levels of *saliva cotinine* (0.71–14.99 ng/mL) were observed in non-smokers. After a mean follow-up of 8 years, these were associated with all-cause deaths (age adjusted hazard ratio [HR] 1.25 [95% CI 1.02–1.53)] and cardiovascular deaths (adj HR 1.21 [95% CI 0.85–1.73)]. High SHS was also associated with elevated C-reactive protein (CRP), which contributes to explaining the association between SHS and deaths due to cardiovascular disease (CVD) [113].

Admission *serum cotinine* concentrations were measured in consecutive patients admitted to nine Scottish hospitals over 23 months. Among 1,261 never smokers, within 30 days, 50 (4%) had died and 35 (3%) had had a non-fatal myocardial infarction. All-cause death increased from 2.1% in those with cotinine ≤0.10 ng/mL to 7.5% in those with cotinine >0.9 ng/mL (χ^2^ test for trend p < 0.001). This difference persisted after adjustment for confounders (adj OR for cotinine >0.9 ng/mL = 4.80 [95% CI 1.96–11.83]; p = 0.003). The same trend was observed for death, cardiovascular death, or myocardial infarction. SHS exposure is thus associated with worse early prognosis after acute coronary syndrome [114].

From the 10-month period preceding the passage of the Scottish smoking ban (2006) to the same period the next year, among all patients admitted with acute coronary syndrome in nine hospitals, there was a decrease in the geometric mean concentrations of *serum cotinine* among never smokers from 0.68 ng/mL to 0.56 ng/mL (p < 0.001), and a 21% reduction in the number of admissions for acute coronary syndrome, together with a self-reported decrease in the weekly duration of exposure to SHS. Reductions in admissions for acute coronary syndrome were also observed among former smokers (19%) and smokers (14%), while the number of deaths among patients with acute coronary syndrome who were not admitted to the hospital decreased by 6%. These reductions contrast with the smaller overall reduction in hospital admissions of 4% in England (where such legislation did not exist) during the same period, and with the 3% mean annual decrease in admissions in Scotland during the decade preceding the study [115].

In Rome, Italy, acute coronary events were compared between the pre- and post-legislation period, taking into account several time-related potential confounders, including *PM_10_* *air pollution*. The PM_10_ concentration decreased, but not significantly, between the year before and after the ban. The age-standardized rate ratios of acute coronary events (defined as hospitalization records of acute coronary events + other out of hospital deaths due to ischemic heart disease from the mortality register) were 0.89 (95% CI 0.85–0.93) post-smoking ban *versus* pre-smoking in the 35–64 years age group and 0.92 (95% CI 0.88–0.97) in the 65–74 years age group, but 1.02 (95% CI 0.98–1.07) in the 75–84 years age group. After simultaneous adjustment for both time trends and hospitalization rates, the results become borderline in the youngest age group (RR 0.94; 95% CI 0.89–1.01) but remain significant in the 65–74 olds (RR 0.90; 95% CI 0.84–0.96). It remains uncertain whether these limited effects (11.2% reduction in the population aged 35–64 years and 7.9% in those aged 65–74 years) are effectively linked with the smoking ban or with the decrease in active smoking suggested by the reduction in cigarette sales in 2005, or with other, perhaps not controlled factors. The level of implementation of the law was not discussed in this study [116].

In US, the cross-sectional relation between SHS exposure and recognized biomarkers of heart disease risk was investigated among 5,599 never smoking adults (serum cotinine levels ≤15 ng/mL) from the third (1988–1991; 1991–1994) national representative examination survey. Serum cotinine (threshold of detection 0.05 ng/mL) was not detectable in 18% of the subjects; the other subjects were distributed into low (0.05–0.215 ng/mL) and high cotinine levels (0.215–15 ng/mL). Levels of *fibrinogen and homocystein* were significantly higher in people with detectable *serum cotinine versus* those without (9–10 mg/dL, p = 0.03 and 0.8 μmol/L, p < 0.001) higher respectively, but were not statistically different between low and high cotinine levels. White blood cell (WBC) counts decreased only for high cotinine levels. CRP did not increase in relation to cotinine. This lack of association between cotinine and CRP could be attributed to the low sensitivity laboratory method used for CRP in this survey. Among 4,990 current active smokers, an adjusted mean increase of 29.2 mg/dL of *fibrinogen* and 1.8 μmol/L of *homocystein* was observed in comparison with never smokers without detectable cotinine. The apparent effects of SHS on fibrinogen and homocystein, markers of cardiovascular risk, in non-smokers were approximately one third to one half those observed in active smokers, while cotinine levels of passive smokers were only ≈ 0.1% of those of active smokers. These disproportionate associations fit the epidemiological evidence of a similar disproportionate association of SHS exposure and active smoking with coronary heart disease risk. SHS is thus an important avoidable cause of cardiovascular disease [117]

In the US NHANES (1999–2002), in non-smoking children and adolescents aged 6–18 years, multiple regression analysis indicated that a change in *serum cotinine* level of 0.5 ng/mL was associated with a 0.96 mg/dL change in CRP (95% CI 0.93–1.00) even after adjustment. There is thus a significant association between serum cotinine and elevated serum CRP [118].

### 5.2. Respiratory Symptoms and Diseases in Adults

Experimental exposure to one hour SHS (set at bar/restaurant levels) by 16 voluntary non-smoking adults is followed by a mean decrease in FEV_1_ (forced expiratory volume in one second) from 4.3 L to 3.8 L and of FEV_1_/FVC (forced vital capacity) from 0.9 to 0.8 (p < 0.05) immediately after exposure, but by a return to baseline values after 3 hours. *Serum cotinine* increased from 8.9 ng/mL before exposure to 35.5 ng/mL 3 hours after exposure. This lag between spirometric and cotinine data is due to the duration of metabolism from nicotine to cotinine. Other inflammatory cytokines are also increased after 3 hours in contrast with the return to the initial spirometric values at that moment [119].

The impact of smoking policy on respiratory health was assessed among food and beverage servers residing in the Vancouver (Canada) area. Acute and chronic respiratory symptoms were the most prevalent where smoking was allowed, less prevalent where smoking was partially restricted and still less prevalent where it was restricted in 100% of the workplaces. The FEV_1_/FVC ratio was lower (77.8%) in facilities where smoking was permitted than where it was prohibited (81.1%) (p = 0.04). In a parallel fashion with this clinical evolution, in a subset of 88 workers, the geometric mean of *hair cotinine* was 1.4 ng/mg for those working in facilities where smoking was prohibited, 4.6 ng/mg with partial restriction and 5.4 ng/mg with no restriction (p = 0.01). In the small subset of workers who underwent spirometric and cotinine measurements, there was no relationship between lung spirometry and hair nicotine levels [120].

In public places in Scotland, concurrently with a decrease in *serum cotinine* levels in 77 non-smoking bar workers, the prevalence of respiratory and sensory symptoms decreased from 79.2% before the smoke-free policy to 53.2% one month after the ban (change 82%; p < 0.001) and to 46.8% 2 months after. FEV_1_ increased from 96.6% predicted to 104.8% predicted after 1 month and to 101.7% after 2 months (change 5.1%; p = 0.002). Among the asthmatic bar workers, exhaled nitric oxide, a marker of inflammation, decreased from 34.3 parts per billion (ppb) to 27.4 ppb one month after the ban (an 0.8-fold change; *p* = 0.04). Smoke-free legislation is thus associated with early improvements in symptoms, minimal increases in spirometric values and decrease of inflammation [121].

Full respiratory function tests were performed on 73 barmen before and after the Irish smoking ban. Among non-smokers, even if significant, the postban increase in flow value measurements remained very limited, and the diffusion capacity for carbon monoxide (DL_CO_), as expected, remained unchanged, while among current smokers, practically all parameters were not significantly different between pre- and post-ban periods. The decrease in prevalence of cough and phlegm was significant and relevant in non-smokers, but not in current smokers. Symptoms and spirometric values evolve concurrently with saliva cotinine measurements [85].

In a longitudinal study of a cohort of 77 non-smoking adults with chronic obstructive pulmonary disease (COPD) in the US, higher levels of SHS exposure, as measured by *urine cotinine,* were marginally associated with worse COPD severity (mean score increment 4.7 points [95% CI −0.1–9.4; p = 0.054]). SHS exposure thus seems to aggravate the symptoms of COPD, even after cessation of active smoking [122].

In Scotland, among 148 patients with severe COPD (among them 39 current smokers), the symptom burden was worse in households with increased *PM_2.5_*, especially among current smokers [123].

In Sweden, before the ban, 87% of non-smoking hospitality workers were exposed to *air nicotine* levels of ≥0.5 μg/m^3^, and 22% after the ban, a relative risk of 0.25 (95% CI 0.15–0.41).There was a reduction of ±50% in respiratory and sensory symptoms, but no notable change in lung function [91].

Among 72 current biologically verified non-smokers in a cohort study of COPD, the increased levels of urine *NNAL-to-creatinine ratio* (a marker of long-term SHS) were associated with greater COPD severity (mean score increase 1.7 points, p = 0.0003) for each interquartile increment of NNAL/creatinine ratio, after adjustment for demographic data and past smoking. They were also related to more severe dyspnoea and more restricted activity (p ≤ 0.05). NNAL, frequently used as a marker of lung carcinogenesis, can thus also be a good long-term biomarker of SHS, showing greater impact on the severity of COPD than cotinine, a short-term biomarker [124].

In Spain [105], 12 months after the smoking ban, concurrently with a decrease in *saliva cotinine* and hours of SHS exposure, the prevalence of cough and phlegm considered together decreased from 40.6% to 15.6% (p < 0.05) in totally non-smoking venues. The prevalence of any respiratory symptoms also decreased post-ban in completely smoke-free venues (from 56.2 to 38.1; p = 0.012), but not where smoking was allowed in some (p = 0.625) or all (p = 0.774) of the premises. In Portugal and Andorra, with no smoking ban, the prevalence of any respiratory symptoms was not significantly different between the two periods (p = 0.07). This study shows that in Spanish venues where smoking was allowed (at least in theory) in physically separated areas, workers still complained of respiratory symptoms. Even in venues where smoking was totally banned, despite a major decrease, the median saliva cotinine concentration (0.50 ng/mL) was still above the acceptable levels of risk (*i.e.*, 0.14 ng/mL [125]).

### 5.3. Low Respiratory Illnesses in Infancy and Early Childhood

In the 2006 US Surgeon General Report, among the large number of papers allowing a causal relationship to be inferred between SHS exposure after birth and lower respiratory illness in infants and children, middle ear disease in children, respiratory symptoms among children of school age, ever having asthma among children of school age and a lower level of lung function during childhood, most concern self-report exposures and only a minority refer to air or biomarkers of exposure [2].

Recent publications show a relationship between biomarkers, asthma and co-morbidities among young asthma patients. In a low-socioeconomic UK community based cross-sectional study, *salivary cotinine* levels were significantly increased in children with doctor-diagnosed asthma compared to those without (p = 0.002); cotinine validated exposure levels were also associated with doctor validated asthma (Adj OR 1.8; CI 1.4–2.5) [126].

Among 222 children in whom asthma was diagnosed by the primary care giver, SHS exposure in and outside the home was validated by *cotinine in serum and hair*, while the level of PM_5_ was measured at home. Despite lower reported exposure to SHS at home (14.9 *vs.* 18.7 CPD), with lower levels of PM_5_ in houses, the levels of serum and hair cotinine were significantly higher among African-American children than among white children (1.41 ng/mL *vs.* 0.97 and 0.25 *vs.* 0.07 ng/mg, respectively), a difference only explained partially by housing volume [127]. The presence of a strict household smoking ban (confirmed by child cotinine assays) vastly reduced SHS exposure in 91 children with asthma matched with 91 healthy children [128].

Among children with asthma, exposure to SHS (measured as serum cotinine levels) was significantly associated with sleep problems (longer sleep-onset delay, sleep-disordered breathing (p = 0.02), parasomnias (p = 0.002) and overall sleep disturbance (p = 0.0002) [129].

Among 200 asthmatic children enrolled in an asthma program, log *salivary cotinine* level was independently associated with externalizing (p = 0.04), headstrong (p = 0.04) and antisocial behavior (p = 0.04) subscales of a 28-item behavior problem index [130]. Among 220 asthmatic children enrolled in an asthma trial, child behavior problems (externalizing, *i.e.*, hyperactivity and aggression, and internalizing, *i.e.*, depression and behavior symptoms) increased with increased SHS exposure measured as *serum cotinine*, but the increase was significant only in boys [131].

### 5.4. Fertility and Pregnancy

For issues concerning female fertility or fecundability, spontaneous abortion, preterm delivery, congenital malformations, cognitive and behavioral development of the infant, height and growth, childhood cancers, leukemias, and lymphomas, the evidence was considered inadequate or only suggestive of a causal relationship with exposure to SHS in the 2006 Surgeon General’s Report [2], but only a minority of studies relied on cotinine measures, and the latter with discordant results.

In Sweden, a population-based case-control study was conducted between 1996 and 1998 comparing exposure to SHS between 463 women with spontaneous abortion and 864 pregnant women as controls. The Adj OR of spontaneous abortion among SHS-exposed women (0.1–15 ng/mL *plasma cotinine*) was 1.67 and that of the active smokers 2.11 in comparison with unexposed non-smokers [132].

Among 441 non-smoking pregnant women, the OR of discontinuation of any breastfeeding after the infant's first 6 months was 2.42 in women whose *blood cotinine* level was above the 75th percentile of cotinine distribution (>0.15 ng/mL) compared with mothers who had lower cotinine levels, with the corresponding OR of discontinuation of full breastfeeding at 1.71 [133].

Among numerous women whose mid-trimester cotinine levels were ≤10 ng/mL (non-smokers), the OR of fetal death and preterm delivery were 3.4 and 1.8, respectively, in the highest cotinine quintile (0.236–10 ng/mL) compared with the lowest quintile (<0.026 ng/mL) [134].

The risk of preterm delivery (<37 weeks) was higher in a group of non-smoking women whose *hair nicotine* after delivery was >4.00 μg/g (Adj OR 6.12) in comparison with those with the lowest hair nicotine concentration (<0.75 μg/g) [135].

### 5.5. Sudden Infant Death Syndrome

In the 2006 Report of the Surgeon General, the evidence was considered sufficient to confer a causal relationship between postnatal exposure to SHS and sudden infant death syndrome [2].

### 5.6. Cancers

In an European case-control study in non-smokers (190 controls and 149 cases, mostly all types of cancers), 4 ABP-Hb adducts, markers of carcinogenicity measured 7 years before the disease (median), were somewhat higher in cases than in controls [136]. In a large nested case-control study within the European prospective investigation into cancer and nutrition (EPIC), in which smoking status was supported by *cotinine measurements*, daily exposure to SHS for many hours during childhood was associated with lung cancer in adulthood (HR 3.63; 95% CI 1.19–11.11) [137].

In addition to these papers, studies showing elevated levels of PPAH-gluc in the air of venues where smoking was allowed [9] or NNAL or NNAL-gluc, benzene and 1–3 butadiene in body fluids of non-smokers [34,36,63,70,78,104], all markers of carcinogenicity, support the biological plausibility of the influence of SHS on the development of cancers, earlier demonstrated in epidemiological studies.

### 5.7. Mental Health

In a representative sample of 5,560 biologically validated (saliva cotinine < 15 μg/L) non-smoking adults without a history of mental illness drawn from the 1998 and 2003 Scottish Health Surveys, higher *saliva cotinine* levels (>0.70 μg/L) were associated with higher odds of psychological distress (OR 1.49; 95% CI 1.13–1.97) in comparison with participants with cotinine levels below the limit of detection (≤0.05 μg/L). In the prospective analysis, the risk of psychiatric hospital admission was related to high SHS exposures (multivariable adjusted HR 2.84; 95% CI 1.07–7.59) [138].

The trend of cognitive impairment (assessed by the lowest 10% of scores in a battery of neuropsychological tests) in a cross-sectional analysis of a national population based survey of 4,809 validated non-smokers in England, was positively related with increasing concentrations of *saliva cotinine* (p for trend 0.02) after adjustment for other risk factors for cognitive impairment. The pattern of association was the same for never smokers and former smokers [139].

### 5.8. Genotoxicity of SHS

The issue of the genotoxicity of SHS was addressed in a recent review of human studies. Biomarker studies carried out among SHS-exposed non-smoking adults show the presence of *metabolites of carcinogens* in urine, of *DNA and/or protein adducts* in peripheral blood or other non-malignant tissues, and the presence of *micronuclei* in bone marrow or peripheral blood. There is thus abundant evidence of the genotoxicity of SHS [140].

## 6. Limitations

The following limitations can be identified in the mentioned studies:

1. The biological differentiation between active non daily smokers and passive smokers remains critical, due to overlapping levels of biomarkers, and particularly short-term biomarkers. The “validated” definition of smokers, sometimes based on different cutoff levels of *plasma cotinine*, is a limitation for the validity of comparisons.
2. Some of the studies can be biased through the use of “convenience samples” instead of random representative sampling. Participants in convenience samples may be more conscious of the SHS problems and more prone to implement the regulations [92,105]. The statistical power in some studies was limited [100].
3. The results of some studies were weakened by the low level of participation and/or by attrition during the process of inclusion or during follow-up [85,88,100,101].
4. The multiple sources of SHS (also outside the workplace and home) are sometimes unrecognized and can have a confounding effect that can impair the exact quantification of differences in markers between different areas or periods [69]. The prevalence of active smoking can also influence the evolution of SHS exposure [90]. Nicotine and cotinine biomarkers can also be influenced by Nicotine Replacement Therapy.
5. The level of compliance with the regulations is not always physically controlled and can play a role on the quantified values observed in pre-and post-ban studies or in contemporaneous comparisons between different sites.

## 7. Conclusions

Earlier and principally recent SHS publications on air and biomarkers objectively:

1. can validate the *smoking status* obtained via self-report (active daily smoker *versus* non-smoker) [1–3], measure the percentages of inexact self-reporting in different circumstances and show the possibility of understatement of SHS exposures in some self-reports [84] (e.g., pregnancy) [77].
2. confirm the *toxin intake* difference between non-smokers not exposed (<0.05 ng/mL serum cotinine) and non-smokers exposed to SHS (<3 ng/mL serum cotinine) and thus the biological plausibility of the earlier epidemiological observations, a essential element for a causal link between SHS and disease [16]; the cut-off between passive and active smokers sometimes shifts towards larger values in periods or environments with higher SHS levels [15,105].
3. *quantify* the level of *exposure* to air smoke toxicants through air markers and their intake through biomarkers in various locations and conditions and show its increase with the duration of exposure. Both are very different in various areas and both are the foundation for regulations and bans [42,45,104].
4. show the possibility of *“third hand smoke”* due to sorption and desorption of nicotine from indoor surfaces, *etc.*, while a limited contribution to SHS can result from *retention of smoke in* the *lungs* and its expiration after the last puffs [54] and also from infiltration from neighboring units [55] or from outdoor air [92].
5. show the deficiencies of *mechanical measures* in controlling SHS (e.g., ventilation, non-physically separated areas for smokers and non-smokers) [10,44,46,83].
6. objectify the control of SHS obtained by the implementation of *total bans* of smoking in public places, and the resulting reduction in prevalence of exposure in many Western countries [9,10,58,83,84,86,88,95,97,98,103,107,108], without a corresponding increase in SHS exposure outside the workplace [109].
7. show in some countries the good *level of implementation* of the bans [87,88,90,100,106,108], which was unexpectedly satisfactory, even in some Mediterranean countries [93], but as the implementation sometimes decreases with time, also the necessity of its persistent control.
8. demonstrate that *partial bans* reduce SHS exposure much less than total bans and that stringent legislation is needed (e.g., bans of smoking rooms, of smoking in terraces outside cafes or restaurants whether covered or not, in airports, in discotheques, *etc.*) [41,43,64,68,92,99,105,110].
9. show that in the *private domain* (home), currently the main location of exposure to SHS, even if it is decreasing [72] (but not everywhere [109]), individual voluntary measures of protection are needed and complete control is obtained only with a total non-smoking policy at home or, but with lower efficacy, by allowing smoking exclusively outside the home, in order to protect children, who are particularly sensitive to SHS [53–55,75,79,111]. In cars, levels of nicotine and PM_2.5_ are high and not fully controlled by ventilation. They remain elevated even in secondhand cars sold by former smoking owners [47–50].
10. demonstrate some *health effects of SHS*. All cause- and cardiovascular deaths are linked to cotinine levels [113,114]. The prognosis of coronary heart disease is negatively influenced by the levels of cotinine due to SHS exposure [115], evolving together with the levels of fibrinogen, homocystein and CRP (all correlated with the coronary risk) [113,118]. The increases in those markers of cardiovascular risk are much larger than the increase in cotinine. This can explain why the difference in coronary risk between passive and active smokers is much lower than what could be expected from their differences in toxin intake [117]. SHS exposure is thus an important avoidable cause of cardiovascular disease.

Negative short-term effects of experimental exposure to SHS [119] and of chronic SHS exposure [120] on respiratory functions are observed, but the minimal positive influence of a smoking ban on spirometric values is controversial, while a decrease in cough and phlegm is more evident [91,105,121] at least in non-smokers [85]. In established COPD, the severity of symptoms is increased by SHS exposure [122–124].

Spontaneous abortions are more frequent among SHS-exposed non-smoking pregnant women than among unexposed non-smokers [132]. The prevalence of fetal death and preterm delivery are linked with biomarkers of exposure [134,135]. The early discontinuation of breastfeeding is more frequent when blood cotinine levels are in the range of SHS exposure [133]. The link between sudden infant death syndrome and exposure to SHS was already demonstrated by earlier studies [2].

There is limited biologically validated evidence of the influence of SHS on cancer prevalence [136], due to the long delay between exposure and disease, but the observed increase in biomarkers of carcinogenicity [34,36,63,70,78,104] demonstrate the biological plausibility of a link between SHS and cancers, previously observed in epidemiological studies.

Higher odds of psychological distress [138] and of cognitive impairment [139] are also associated with increased cotinine levels. Genotoxicity of SHS is shown by the presence of carcinogen metabolites and of DNA adducts in blood and other tissues and by the presence of micronuclei in bone marrow or peripheral blood of non-smokers exposed to SHS [140].

In spite of the above mentioned limitations, given the consistency of the recent results (as opposed to some contradictions in the earlier periods), there is overwhelming evidence of local variations in exposure to SHS, of reductions in exposure after recent smoking bans, and of the effects of SHS on health parameters.

## References

[1] U.S. Department of Health and Human Services (1986). The Health Consequences of Involuntary Smoking: A Report of the Surgeon General.

[2] US Department of Health and Human Services (2006). The Health Consequences of Involuntary Exposure to Tobacco Smoke: A Report of the Surgeon General.

[3] National Cancer Institute (1999). Health Effects of Exposure to Environmental Tobacco Smoke: The Report of California Environmental Protection Agency.

[4] Callinan JE, Clarke A, Doherry K, Kelleher C (2010). Legislative smoking bans for reducing secondhand smoke exposure, smoking prevalence and tobacco consumption. Cochrane Database Syst Rev.

[5] Directive 2000/69/EC of the European Parliament and of the Council of 16 November 2000 (2000). Relating to limit values for benzene and carbon monoxide in ambient air. Off. J. Eur. Commun.

[6] Daisey JM (1999). Tracers for assessing exposure to environmental tobacco smoke: What are they tracing?. Environ. Health Perspect.

[7] (2005). WHO Air Quality Guidelines for Particulate Matter, Ozone, Nitrogen Dioxide and Sulfur Dioxide Global Update 2005 Summary of Risk Assessment.

[8] Repace J, Al-Delaimy WK, Bernert JT (2006). Correlating atmospheric and biological markers in studies of secondhand tobacco smoke exposure and dose in children and adults. J. Occup. Environ. Med.

[9] Repace J (2004). Respirable particles and carcinogens in the air of Delaware hospitality venues before and after a smoking ban. J. Occup. Environ. Med.

[10] Repace JL, Hyde JN, Brugge D (2006). Air pollution in Boston bars before and after a smoking ban. BMC Public Health.

[11] Al-Delaimy WK, Crane J, Woodward A (2002). Is the hair nicotine level a more accurate biomarker of environmental tobacco smoke exposure than urine cotinine?. J. Epidemiol. Community Health.

[12] Kim SR, Wipfli H, Avila-Tang E, Samet JM, Breysse PN (2009). Method validation for measurement of hair nicotine level in nonsmokers. Biomed. Chromatogr.

[13] Florescu A, Ferrence R, Einarson TR, Selby P, Kramer M, Woodruff S, Grossman L, Rankin A, Jacqz-Aigrain E, Koren G (2007). Reference values for hair cotinine as a biomarker of active and passive smoking in women of reproductive age, pregnant women, children, and neonates: systematic review and meta-analysis. Ther. Drug Monit.

[14] Al-Delaimy WK, Willet WC (2008). Measurement of tobacco smoke exposure: Comparison of toenail nicotine biomarkers and self-reports. Cancer Epidemiol. Biomarkers Prev.

[15] Jarvis MJ, Fidler J, Mindell J, Feyerabend C, West R (2008). Assessing smoking status in children, adolescents and adults: Cotinine cut-points revisited. Addiction.

[16] Benowitz NL, Bernert JT, Caraballo RS, Holiday DB, Wang J (2009). Optimal serum cotinine levels for distinguishing cigarette smokers and nonsmokers with different racial/ethnic groups in the United States between 1999 and 2004. Am. J. Epidemiol.

[17] Jarvis MJ, Russell MAH, Benowitz NL, Feyerabend C (1988). Elimination of cotinine from body fluids: implications of noninvasive measurements of tobacco smoke exposure. Am. J. Public Health.

[18] Curvall M, Elwin C-E, Kazemi-Vala E, Werholm C, Enzell CR (1990). The pharmacokinetics of cotinine in plasma and saliva from non-smoking healthy volunteers. Eur. J. Clin. Pharmacol.

[19] Rose JE, Levin ED, Benowitz N (1993). Saliva nicotine as an index of plasma levels in nicotine skin patch users. Ther. Drug Monit.

[20] Bernert JT, McGuffey JE, Morrison MA, Pirkle JL (2000). Comparison of serum and salivary cotinine measurements by a sensitive high-performance liquid chromatography-tandem mass spectrometry method as an indicator of exposure to tobacco smoke among smokers and nonsmokers. J. Anal. Toxicol.

[21] Jarvis M, Tunstall-Pedoe H, Feyerabend C, Vesey C, Salloojee Y (1984). Biochemical markers of smoke absorption and self reported exposure to passive smoking. J. Epidemiol. Community Health.

[22] Thompson SG, Barlow RD, Wald NJ, van Vunakis H (1990). How should urinary cotinine concentrations be adjusted for urinary creatinine concentrations?. Clin. Chim. Acta.

[23] Benowitz NL (1996). Cotinine as a biomarker of environmental tobacco smoke exposure. Epidemiol. Rev.

[24] Benowitz NL, Dains KM, Dempsey D, Herrera B, Yu L, Jacob P (2009). Urine nicotine metabolite concentrations in relation to plasma cotinine during low-level nicotine exposure. Nicotine Tob. Res.

[25] Matt GE, Hovell MF, Quitana PJE, Zakarian J, Liles S, Meltzer SB, Benowitz NL (2007). The variability of urinary cotinine levels in young children: Implications for measuring ETS exposure. Nicotine Tob. Res.

[26] Bernert JT, Gordon SM, Jain RB, Brinkman MC, Sosnoff CS, Seyler TH, Xia Y, McGuffey JE, Ashley DL, Pirkle JL, Sampson EJ (2009). Increases in tobacco exposure biomarkers measured in non-smokers exposed to sidestream cigarette smoke under controlled conditions. Biomarkers.

[27] (2003). Results from the 2002 National Survey on Drug Use and Health: National Findings.

[28] Benowitz NL, Dains KM, Dempsey D, Yu L, Jacob P (2010). Estimation of nicotine dose after low-level exposure using plasma and urine nicotine metabolites. Cancer Epidemiol. Biomarkers Prev.

[29] Bernert JT, Jacob P, Holiday DB, Benowitz NL, Sosnoff CS, Doig MV, Feyerabend C, Aldous KM, Sharifi M, Kellog MD, Langman LJ (2009). Interlaboratory comparability of serum cotinine measurements at smoker and nonsmoker concentration levels: A round-robin study. Nicotine Tob. Res.

[30] Chen R, Tavendale R, Tunstall-Pedoe H (2002). Measurement of passive smoking in adults: Self-reported questionnaire or serum cotinine?. J. Cancer Epidemiol. Prev.

[31] Polańska K, Hanke W, Laudański T, Kalinka J (2007). Serum cotinine level as a biomarker of tobacco smoke exposure during pregnancy. Ginekol. Pol.

[32] Best D, Green EM, Smith JH, Perry DC (2010). Dipstick tests for secondhand smoke exposure. Nicotine Tob. Res.

[33] Cooke F, Bullen C, Whittaker R, McRobbie H, Chen M-H, Walker N (2008). Diagnostic accuracy of NicAlert cotinine test strips in saliva for verifying smoking status. Nicotine Tob. Res.

[34] Hecht SS (2004). Carcinogen derived biomarkers: Applications in studies of human exposure to secondhand tobacco smoke. Tob. Control.

[35] Gunier RB, Reynolds P, Hurley SE, Yerabati S, Hertz A, Strickland P, Horn-Ross PL (2006). Estimating exposure to polycyclic hydrocarbons: A comparison of survey, biological monitoring, and geographic information system-based methods. Cancer Epidemiol. Biomarkers Prev.

[36] McNabola A, Broderick B, Johnston P, Gill L (2006). Effects of the smoking ban on benzene and 1,3-butanediene levels in pubs in Dublin. J. Environ. Sci. Health A Tox. Hazard Subst. Environ. Eng.

[37] Faught BE, Flouris AD, Cairney J (2009). Epidemiological evidence associating secondhand smoke exposure with cardiovascular disease. Inflamm. Allergy Drug Targets.

[38] Rosen LJ, Zucker D, Rosenberg H, Connolly G (2008). Secondhand smoke in Israeli bars, pubs and cafes. Isr. Med. Assoc. J.

[39] Schneider S, Seibold B, Schunk S, Jentzsch E, Dresler C, Travers MJ, Hyland A, Pötschke-Langer M (2008). Exposure to secondhand smoke in Germany: Air contamination due to smoking in German restaurants, bars, and other venues. Nicotine Tob. Res.

[40] Edwards R, Hasselholdt CP, Hargreaves K, Probert C, Holford R, Hart J, van Tongeren M, Watson AF (2006). Levels of second hand smoke in pubs and bars by deprivation and food-serving status: a cross-sectional study from North West England. BMC Public Health.

[41] Cameron M, Brennan E, Durkin S, Borland R, Travers MJ, Hyland A, Spittal MJ, Wakefield MA (2010). Secondhand smoke exposure (PM_2.5_) in outdoor dining areas and its correlates. Tob. Control.

[42] Lopez MJ, Nebot M, Albertini M, Birkui P, Centrich F, Chudzikova M, Georgouli M, Gorini G, Moshammer H, Mulcahy M, Pilali M, Serrahima E, Tutka P, Fernandez E (2008). Secondhand smoke exposure in hospitality venues in Europe. Environ. Health Perspect.

[43] Nebot M, López MJ, Gorini G, Neuberger M, Axelsson S, Pilali M, Fonseca C, Abdenbi K, Hackshaw A, Moshammer H, Laurent AM, Salles J, Georgouli M, Fondelli MC, Serrahima E, Centrich F, Hammond SK (2005). Environmental tobacco smoke exposure in public places of European cities. Tob. Control.

[44] Navas-Acien A, Peruga A, Breysse P, Zavaleta A, Blanco-Marquizo A, Pitarque R, Acuna M, Jimenez-Reyes K, Colombo VL, Gamarra G, Stillman FA, Samet J (2004). Secondhand tobacco smoke in public places in Latin America, 2002–2003. JAMA.

[45] Hyland A, Travers MJ, Dresler C, Higbee C, Cummings KM (2008). A 32-country comparison of tobacco smoke derived particle levels in indoor public places. Tob. Control.

[46] Repace J (2004). Flying the smoky skies: Secondhand smoke exposure of flight attendants. Tob. Control.

[47] Jones MR, Navas-Acien A, Yuan J, Breysse PN (2009). Secondhand tobacco smoke concentrations in motor vehicles: A pilot study. Tob. Control.

[48] Sendzik T, Fong GT, Travers MJ, Hyland A (2009). An experimental investigation of tobacco smoke pollution in cars. Nicotine Tob. Res.

[49] Vardavas CI, Linardakis M, Kafatos AG (2006). Environmental tobacco smoke exposure in motor vehicles: A preliminary study. Tob. Control.

[50] Matt GE, Quintana PJE, Hovell MF, Chatfield D, Ma DS, Romero R, Uribe A (2008). Residual tobacco smoke pollution in used cars for sale: Air, dust and surfaces. Nicotine Tob. Res.

[51] Siegel M, Skeer M (2003). Exposure to secondhand smoke and excess lung cancer mortality risk among workers in the “5B’s”: Bars, bowling alleys, billiard halls, betting establishments and bingo parlours. Tob. Control.

[52] Stillman F, Navas-Acien A, Ma J, Ma S, Avila-Tang E, Breysse P, Yang G, Samet J (2007). Second-hand tobacco smoke in public places in urban and rural China. Tob. Control.

[53] Van Deusen A, Hyland A, Travers MJ, Wang C, Higbee C, King BA, Alford T, Cummings KM (2009). Seconhand smoke and particulate matter exposure in the home. Nicotine Tob. Res.

[54] Invernizzi G, Ruprecht A, De Marco C, Paredi P, Boffi R (2007). Residual tobacco smoke: Measurement of its washout time in the lung and of its contribution to environmental tobacco smoke. Tob. Control.

[55] Kraev TA, Adamkiewicz G, Hammond SK, Spengler JD (2009). Indoor concentrations of nicotine in low-income, multi-unit housing: associations with smoking behaviours and housing characteristics. Tob. Control.

[56] Jedrychowski WA, Perera FP, Pac A, Jacek R, Whyatt RM, Spengler JD, Dumyahn TS, Sochacka-Tatara E (2006). Variability of total exposure to PM_2.5_ related to indoor and outdoor pollution sources Krakow study in pregnant women. Sci Total Environ.

[57] Maziak W, Rastam S, Ibrahim I, Ward KD, Eissenberg T (2008). Waterpipe-associated particulate matter emissions. Nicotine Tob. Res.

[58] Centers for Disease Control and Prevention (CDC) (2008). Disparities in secondhand smoke exposure—United States, 1988–1994 and 1999–2004. MMWR Morb. Mortal. Wkly. Rep.

[59] Centers for Disease Control and Prevention (CDC) (2010). Vital signs: Nonsmokers’ exposure to secondhand smoke—United States, 1999–2008. MMWR Morb. Mortal. Wkly. Rep.

[60] Wortley PM, Caraballo RS, Pederson LL, Pechacek TF (2002). Exposure to secondhand smoke in the workplace: Serum cotinine by occupation. J. Occup. Environ. Med.

[61] Ellis JA, Gwynn C, Garg RK, Philburn R, Aldous KM, Perl SB, Thorpe L, Frieden TR (2009). Seconhand smoke exposure among nonsmokers nationally and in New York City. Nicotine Tob. Res.

[62] Perera FP, Illman SM, Kinney PL, Whyatt RM, Kelvin EA, Shepard P, Evans D, Fullilove M, Ford J, Miller RL, Meyer IH, Rauh VA (2002). The challenge of preventive environmentally related disease in young children: community-based research in New York City. Environ. Health Perspect.

[63] Tulunay OE, Hecht SS, Carmella SG, Zhang Y, Lemmonds C, Murphy S, Hatsukami DK (2005). Urinary metabolites of a tobacco-specific lung carcinogen in nonsmoking hospitality workers. Cancer Epidemiol. Biomarker. Prev.

[64] Bates MN, Fawcett J, Dickson S, Berezowski R, Garrett N (2002). Exposure of hospitality workers to environmental tobacco smoke. Tob. Control.

[65] Repace J, Hughes E, Benowitz N (2006). Exposure to second-hand smoke air pollution assessed from bar patrons’ urinary cotinine. Nicotine Tob. Res.

[66] Fowles J, Christophersen A, Fernando D, Lea R, Woodward A, Dickson S, Hosking M, Berezowski R (2006). Secondhand tobacco smoke exposure in New Zealand bars: Results prior to implementation of the bar smoking ban. N. Z. Med. J.

[67] Hedley AJ, McGhee SM, Repace JL, Wong LC, Yu MY, Wong TW, Lam TH (2006). Risks for heart disease and lung cancer from passive smoking by workers in the catering industry. Toxicol. Sci.

[68] Lazcano-Ponce E, Benowitz N, Sanchez-Zamorano LM, Barbosa-Sanchez L, Valdes-Salgado R, Jacob P, Diaz R, Hernandez-Avila M (2007). Secondhand smoke exposure in Mexican discotheques. Nicotine Tob. Res.

[69] Okoli CTC, Hall LA, Rayens MK, Hahn EJ (2007). Measuring tobacco smoke exposure among smoking and nonsmoking bar and restaurants Workers. Biol. Res. Nurs.

[70] Anderson KE, Kliris J, Murphy L, Carmella SG, Han S, Link C, Bliss RL, Puumala S, Murphy SE, Hecht SS (2003). Metabolites of a tobacco-specific lung carcinogen in nonsmoking casino patrons. Cancer Epidemiol. Biomarker. Prev.

[71] Benowitz NL, Schultz KE, Haller CA, Wu AH, Dains KM, Jacob P (2009). Prevalence of smoking assessed biochemically in an urban public hospital: A rationale for routine cotinine screening. Am. J. Epidemiol.

[72] Jarvis MJ, Mindell J, Gilmore A, Feyerabend C, West R (2009). Smoke-free homes in England: Prevalence, trends and validation by cotinine in children. Tob. Control.

[73] Polańska K, Hanke W, Sobala W, Ligocka D (2009). Prenatal and postnatal child exposure to environmental tobacco smoke. Przegl. Lek.

[74] Puig C, Garcia-Algar O, Monleon T, Pacifici R, Zuccaro P, Sunyer J, Figueroa C, Pichini C, Vall O (2008). A longitudinal study of environmental tobacco smoke exposure in children: Parental self reports *versus* age dependent biomarkers. BMC Public Health.

[75] Groner J, Wadwa P, Hoshaw-Woodard S, Hayes J, Klein J, Koren G, Castile RG (2004). Active and passive tobacco smoke exposure: A comparison of maternal and child hair cotinine levels. Nicotine Tob. Res.

[76] Yoo S-H, Paek Y-J, Kim S-S, Lee D-H, Seo D-K, Seong M-W, Chang H-M, Choi S-T, Im H-J (2010). Hair nicotine levels in non-smoking pregnant women whose spouses smoke outside of the home. Tob. Control.

[77] de Chazeron I, Llorca P-M, Ughetto S, Coudore F, Boussiron D, Perriot J, Vendittelli F, Sapin V, Lemery D (2007). Occult maternal exposure to environmental tobacco smoke exposure. Tob. Control.

[78] Hecht SS, Carmella SG, Le K-A, Murphy SE, Boettcher AJ, Le C, Koopmeiners J, An L, Hennrikus DJ (2006). 4-(methylnitrosamino)-1-(3-pyridyl)-1-butanol and its glucuronides in the urine of infants exposed to environmental tobacco smoke. Cancer Epidemiol. Biomarkers Prev.

[79] Seong MW, Hwang JH, Moon JS, Ryu HJ, Kong SY, Um TH, Park JG, Lee DH (2008). Neonatal hair nicotine levels and fetal exposure to paternal smoking at home. Am. J. Epidemiol.

[80] Hovell MF, Zakarian JM, Matt GE, Liles S, Jones JA, Hofstetter R, Larson SN, Benowitz NL (2009). Counseling to reduce children’s secondhand smoke exposure and help parents quit smoking: A controlled trial. Nicotine Tob. Res.

[81] Roza MR, Viegas CAS (2007). The dog as a passive smoker: Effects of exposure to environmental cigarette smoke on domestic dogs. Nicotine Tob. Res.

[82] McNiel EA, Carmella SG, Heath LA, Bliss RL, Le KA, Hecht SS (2007). Urinary biomarkers to assess exposure of cats to environmental tobacco smoke. Am. J. Vet. Res.

[83] Centers for Disease Control and Prevention (CDC) (2004). Indoor air quality in hospitality venues before and after implementation of a clean indoor air law—Western New York, 2003. MMWR Morb. Mortal. Wkly. Rep.

[84] Mulcahy M, Evans DS, Hammond SK, Repace JL, Byrne M (2005). Secondhand smoke exposure and risk following the Irish smoking ban: An assessment of salivary cotinine concentrations in hotel workers and air nicotine levels in bars. Tob. Control.

[85] Goodman P, Agnew M, McCaffrey M, Paul G, Clancy L (2007). Effects of the Irish smoking ban on respiratory health of bar workers and air quality in Dublin pubs. Am. J. Respir. Crit. Care Med.

[86] Gotz NK, van Tongeren M, Wareing H, Wallace LM, Semple S, MacCalman L (2008). Changes in air quality and second-hand smoke exposure in hospitality sector businesses after introduction of the English smoke-free legislation. J. Public Health.

[87] Semple S, Creely KS, Naji A, Miller BG, Ayres JG (2007). Secondhand smoke levels in Scottish pubs: The effect of smoke-free legislation. Tob. Control.

[88] Semple S, Maccalman L, Naji AA, Dempsey S, Hilton S, Miller BG, Ayres JG (2007). Bar workers’ exposure to secondhand smoke: The effect of Scottish smoke-free legislation on occupational exposure. Ann. Occup. Hyg.

[89] Johnsson T, Tuomi T, Riuttala H, Hyvärinen M, Rothberg M, Reijula K (2006). Environmental tobacco smoke in Finnish Restaurants and bars before and after smoking restrictions were introduced. Ann. Occup. Hyg.

[90] Ellingsen DG, Fladseth G, Daae HL, Gjøstad M, Kjaerheim K, Skogstad M, Olsen R, Thorud R, Molander P (2006). Airborne exposure and biological monitoring of bar and restaurant workers before and after the introduction of smoking ban. J. Environ. Monit.

[91] Larsson M, Boëthius G, Axelsson S, Montgomery SM (2008). Exposure to environmental tobacco smoke and health effects among hospitality workers in Sweden—before and after the implementation of a smoke-free law. Scand. J. Work Environ. Health.

[92] Brennan E, Cameron M, Warne C, Durkin S, Borland R, Travers MJ, Hyland A, Wakefield MA (2010). Secondhand smoke drift: Examining the influence of indoor smoking bans on indoor and outdoor air quality at pubs and bars. Nicotine Tob. Res.

[93] Valente P, Forastiere F, Bacosi A, Cattani G, Di Carlo S, Ferri M, Figà-Talamanca I, Marconi A, Paoletti L, Perucci C, Zuccaro P (2007). Exposure to fine and ultrafine particles from secondhand smoke in public places before and after the smoking ban, Italy 2005. Tob. Control.

[94] Proescholdbell SK, Foley KL, Johnson J, Malek SH (2008). Indoor air quality in prisons before and after implementation of a smoking ban law. Tob. Control.

[95] Heloma A, Jaakkola MS (2003). Four-year follow-up of smoke exposure, attitudes and smoking behavior following enactment of Finland’s national smoke-free work-place law. Addiction.

[96] Blanco-Marquizo A, Goja B, Peruga A, Jones MR, Yuan J, Samet JM, Breysse PN, Navas-Acien A (2010). Reduction of secondhand tobacco smoke in public places following national smoke-free legislation in Uruguay. Tob. Control.

[97] Gorini G, Moshammer H, Sbrogiò L, Gasparrini A, Nebot M, Neuberger M, Tamang E, Lopez MJ, Galeone D, Serrahima E (2008). Italy & Austria before and after study Working Group. Italy and Austria before and after study: Second-hand smoke exposure in hospitality premises before and after 2 years from the introduction of the Italian smoking ban. Indoor Air.

[98] Centers for Disease Control and Prevention (CDC) (2007). Reduced secondhand smoke exposure after implementation of a comprehensive statewide smoking ban—New York, June 26, 2003–June 30, 2004. MMWR Morb. Mortal. Wkly. Rep.

[99] Pickett MS, Schober SE, Brody DJ, Curtin LR, Giovino GA (2006). Smoke-free laws and secondhand smoke exposure in US non-smoking adults, 1999–2002. Tob. Control.

[100] Farrelly MC, Nonnemaker JM, Chou R, Hyland A, Peterson KK, Bauer UE (2005). Changes in hospitality workers’ exposure to secondhand smoke following the implementation of New York’s smoke-free law. Tob. Control.

[101] Hahn EJ, Rayens MK, York N, Okoli CT, Zhang M, Dignan M, Al-Delaimy WK (2006). Effects of a smoke-free law on hair nicotine and respiratory symptoms of restaurant and bar workers. J. Occup. Environ. Med.

[102] Jensen JA, Schillo BA, Moilanen MM, Lindgren BR, Murphy S, Carmella S, Hecht SS, Hatsukami DK (2010). Tobacco smoke exposure in nonsmoking hospitality workers before and after a state smoking ban. Cancer Epidemiol. Biomarker. Prev.

[103] Abrams SM, Mahoney MC, Hyland A, Cummings M, Davis W, Song L (2006). Early evidence on the effectiveness of clean indoor air legislation in New York State. Am. J. Public Health.

[104] Stark MJ, Rohde K, Maher JE, Pizacani BA, Dent CW, Bard R, Carmella SG, Benoit AR, Thomson NM, Hecht SS (2007). The impact of clean indoor air exemptions and preemption policies on the prevalence of a tobacco-specific lung carcinogen among nonsmoking bar and restaurant workers. Am. J. Public Health.

[105] Fernández E, Fu M, Pascual JA, López MJ, Pérez-Rios M, Schiaffino A, Martinez-Sánchez JM, Ariza C, Saltó E, Nebot M (2009). The Spanish Smoking Law Evaluation Group. Impact of the Spanish smoking law on exposure to second-hand smoke and respiratory health in hospitality workers: A Cohort Study. PLoS ONE.

[106] Bondy SJ, Zhang B, Kreiger N, Selby P, Benowitz N, Travis H, Florescu A, Greenspan NR, Ferrence R (2009). Impact of an indoor smoking ban on bar workers’ exposure to secondhand smoke. J. Occup. Environ. Med.

[107] Allwright S, Paul G, Greiner B, Mullally BJ, Pursell B, Kelly A, Bonner B, D’Eath M, McConnell B, McLaughlin JP, O’Donovan D, O’Kane E, Perry IJ (2005). Legislation for smoke-free workplaces and health of bar workers in Ireland: before and after study. BMJ.

[108] Fernando D, Fowles J, Woodward A, Christophersen A, Dickson S, Hosking M, Berezowski R, Lea RA (2007). Legislation reduces exposure to second-hand tobacco smoke in New Zealand bars by about 90%. Tob. Control.

[109] Haw SJ, Gruer L (2007). Changes in exposure of adult non-smokers to secondhand smoke after implementation of smoke-free legislation in Scotland: National cross sectional survey. BMJ.

[110] Dove MS, Dockery DW, Connolly GN (2010). Smoke-free air laws and secondhand smoke exposure among nonsmoking youth. Pediatrics.

[111] Akhtar PC, Currie DB, Currie CE, Haw SJ (2007). Changes in child exposure to environmental tobacco smoke (CHETS) study after implementation of smoke-free legislation in Scotland: National cross sectional survey. BMJ.

[112] Wakefield M, Banham D, McCaul K, Martin J, Ruffin R, Badcock N, Roberts L (2002). Effect of feedback regarding urinary cotinine and brief tailored advice on home smoking restrictions among low-income parents of children with asthma: A controlled trial. Prev. Med.

[113] Hamer M, Stamatakis E, Kivimaki M, Lowe GD, Batty GD (2010). Objectively measured secondhand smoke exposure and risk of cardiovascular disease: What is the mediating role of inflammatory and hemostatic factors?. J. Am. Coll. Cardiol.

[114] Pell JP, Haw S, Cobbe S, Newby DE, Pell AC, Fischbacher C, Pringle S, Murdoch D, Dunn F, Oldroyd K, MacIntyre P, O’Rourke B, Borland W (2009). Secondhand smoke exposure and survival following acute coronary syndrome: Prospective cohort study of 1261 consecutive admissions among never-smokers. Heart.

[115] Pell JP, Haw S, Cobbe S, Newby DE, Pell AC, Fischbacher C, McConnachie A, Pringle S, Murdoch D, Dunn F, Oldroyd K, Macintyre P, O’Rourke B, Borland W (2008). Smoke-free legislation and hospitalizations for acute coronary syndrome. N. Engl. J. Med.

[116] Cesaroni G, Forastiere F, Agabiti N, Valente P, Zuccaro P, Perucci CA (2008). Effect of the Italian smoking ban on population rates of acute coronary events. Circulation.

[117] Venn A, Britton J (2007). Exposure to secondhand smoke and biomarkers of cardiovascular disease risk in never-smoking adults. Circulation.

[118] Wilkinson JD, Lee DJ, Arheart KL (2007). Secondhand smoke exposure and C-reactive protein levels in youth. Nicotine Tob. Res.

[119] Flouris AD, Metsios GS, Carrillo AE, Jamurtas AZ, Gourgoulianis K, Kiropoulos T, Tzatzarakis MN, Tsatsakis AM, Koutedakis Y (2009). Acute and short-term effects of secondhand smoke on lung function and cytokine production. Am. J. Respir. Crit. Care Med.

[120] Dimich-Ward H, Lawson J, Hingston A, Chan-Yeung M (2005). Impact of smoking policy on the respiratory health of food and beverage servers. Scand. J. Work Environ. Health.

[121] Menzies D, Nair A, Williamson PA, Schembri S, Al-Khairalla MZH, Barnes M, Fardon TC, McFarlane L, Magee GJ, Lipworth BJ (2006). Respiratory symptoms, pulmonary function, and markers of inflammation among bar workers before and after a legislative ban on smoking in public places. JAMA.

[122] Eisner MD, Balmes J, Yelin EH, Katz PP, Hammond SK, Benowitz N, Blanc PD (2006). Directly measured secondhand smoke exposure and COPD health. BMC Pulm. Med.

[123] Osman LM, Douglas JG, Garden C, Reglitz K, Lyon J, Gordon S, Ayres JG (2007). Indoor air quality in homes of patients with chronic obstructive pulmonary disease. Am. J. Respir. Crit. Care Med.

[124] Eisner MD, Jacob P, Benowitz NL, Balmes J, Blanc PD (2009). Longer term exposure to secondhand smoke and health outcomes in COPD: Impact of urine 4-(methylnitrosamino)-1- (3-pyridyl)-1-butanol. Nicotine Tob. Res.

[125] Repace JL, Binot J, Bayard S, Emmons K, Hammond SK (1998). Air nicotine and saliva cotinine as indicators of passive exposure and risk. Risk Analysis.

[126] Delpisheh A, Kelly Y, Rizwan S, Brabib BJ (2008). Salivary cotinine, doctor-diagnosed asthma and respiratory symptoms in primary schoolchildren. Matern. Child Health J.

[127] Wilson SE, Kahn RS, Khoury J, Lanphear BP (2005). Racial differences in exposure to environmental tobacco smoke among children. Environ. Health Perspect.

[128] Wamboldt FS, Balkissoon RC, Rankin AE, Szefler SJ, Hammond SK, Glasgow RE, Dickinson WP (2008). Correlates of household smoking bans in low-income families of children with and without asthma. Fam. Process.

[129] Yolton K, Xu Y, Khoury J, Succop P, Lanphear B, Beebe DW, Owens J (2010). Associations between secondhand smoke exposure and sleep patterns in children. Pediatrics.

[130] Fagnano M, Conn KM, Halterman JS (2008). Environmental tobacco smoke and behaviors of inner-city children with asthma. Ambul. Pediatr.

[131] Yolton K, Khoury J, Hornung R, Dietrich K, Succop P, Lanphear B (2008). Environmental tobacco smoke exposure and child behaviors. J. Dev. Behav. Pediatr.

[132] George L, Granath F, Johansson AL, Annerén G, Cnattingius S (2006). Environmental tobacco smoke and risk of spontaneous abortion. Epidemiology.

[133] Jedrychowski W, Perera F, Mroz E, Edwards S, Flak E, Rauh V, Pac A, Budzyn-Mrozek D, Musial A (2008). Prenatal exposure to passive smoking and duration of breastfeeding in nonsmoking women: Krakow inner city prospective cohort study. Arch. Gynecol. Obstet.

[134] Kharrazi M, DeLorenze GN, Kaufman FL, Eskenasi B, Bernert JJ, Graham S, Pearl M, Pirkle J (2004). Environmental tobacco smoke and pregnancy outcome. Epidemiology.

[135] Jaakkola JJ, Jaakkola N, Zahlsen K (2001). Fetal growth and length of gestation in relation to prenatal exposure to environmental tobacco smoke assessed by hair nicotine concentration. Environ. Health Perspect.

[136] Airoldi L, Vineis P, Colombi A, Olgiati L, Dell’Osta C, Fanelli R, Manzi L, Veglia F, Autrup H, Dunning A (2005). 4-aminobiphenyl -hemoglobin adducts and risk of smoking-related disease in never smokers and former smokers in the European Prospective Investigation into Cancer and Nutrition prospective study. Cancer Epidemiol. Biomarker. Prev.

[137] Vineis P, Airoldi L, Veglia F, Olgiati L, Pastorelli R, Autrup H, Dunning A, Garte S, Gormally E, Hainaut P (2005). Environmental tobacco smoke and risk of respiratory cancer and chronic obstructive pulmonary disease in former smokers and never smokers in the EPIX prospective study. BMJ.

[138] Hamer M, Stamatakis E, Batty GD (2010). Objectively assessed secondhand smoke exposure and mental health in adults: Cross-sectional and prospective evidence from the Scottish Health Survey. Arch. Gen. Pychiatry.

[139] Llewellyn DJ, Lang IA, Langa KM, Naughton F, Matthews FE (2009). Exposure to secondhand smoke and cognitive impairment in non-smokers: national cross sectional study with cotinine measurement. BMJ.

[140] Husgafvel-Pursiainen K (2004). Genotoxicity of environmental tobacco smoke: A review. Mutat. Res.

